# Coordinated changes in midkine expression and midkine-associated multiomic profile in glioma microenvironment

**DOI:** 10.1038/s41598-025-16253-5

**Published:** 2025-08-20

**Authors:** Mieszko Lachota, Katarzyna Zielniok, Agata Góźdź, Patrycja Szpak, Ilona Kalaszczyńska, Radosław Zagożdżon

**Affiliations:** 1 Department of Ophthalmology, Children’s Memorial Health Institute, Warsaw, Poland; 2 Laboratory of Cellular and Genetic Therapies, Medical University of Warsaw, Warsaw, Poland; 3 Department of Histology and Embryology, Centre for Biostructure Research, Medical University of Warsaw, Warsaw, Poland

**Keywords:** Midkine, MDK, Glioblastoma, GBM, Glioma, Tumor microenvironment, TME, TCGA, CNS cancer, Cytokines

## Abstract

Midkine (MDK), a multifunctional growth factor, has been implicated in promoting tumor progression, yet its role in glioblastoma (GBM) remains insufficiently characterized. To investigate MDK’s function in glioma, we integrated four RNA-Seq datasets into a harmonized cohort of 1,017 adult gliomas, including 256 GBM samples. We complemented this with freshly collected human GBM tissues and matched primary cell cultures to evaluate MDK expression and secretion patterns, further contextualized using single-cell RNA-Seq. Finally, we tested the impact of GBM-derived MDK on macrophage secretome composition to validate our in silico observations. We found that *MDK* expression increases with tumor grade in *IDH*^wildtype^ gliomas, accompanied by a shift in isoform proportions favoring the canonical *MDK* transcript. High *MDK* expression was associated with poor prognosis specifically in GBM, where the *MDK*^high^ subgroup comprised 75% of cases. *MDK*^high^ GBMs exhibited a distinctive multiomic signature, including elevated chemokine and cytokine expression. Functionally, GBM-derived MDK induced macrophages to secrete multiple cytokines and chemokines, suggesting its role in reshaping the tumor microenvironment. Our findings reveal MDK’s previously underappreciated role in GBM aggressiveness and immune modulation, underscoring its potential as a biomarker and actionable therapeutic target for most GBM patients.

**Supplementary Information:**

The online version contains supplementary material available at 10.1038/s41598-025-16253-5.

## Introduction

Glioblastoma (GBM) is the most common malignant glial tumor in adults and the leading cause of cancer deaths among patients aged 15 to 34^1,2^. According to the 2021 World Health Organization (WHO) Classification of Gliomas, adult-type diffuse astrocytic tumors are grouped based on isocitrate dehydrogenase (*IDH*) mutation status, with only *IDH*^wildtype^ tumors with either characteristic morphology or molecular features of glioblastoma being classified as “glioblastoma, *IDH*^wildtype^"^3^. First-line treatment, consisting of radical and safe resection supported by chemoradiotherapy, yields a median overall survival (OS) of approximately 16 months, which has not improved since 1990s^1,2,4^. There is no standard second-line therapy to prolong OS, with virtually inevitable recurrence less than a year after surgery^1,2^. While for many systemic cancers, targeted treatments became part of the standard of care, in grade IV *IDH*^wildtype^ gliomas (GBM), the clinical benefit is only seen in 1–2% of patients with v-Raf murine sarcoma viral oncogene homolog B1 (BRAF) mutations treated with *BRAF* p.V600E inhibitors^5,6^. There is a pressing need for new targeted therapies and biomarkers that could allow for early detection and identification of patients benefiting from additional targeted treatment. Despite the advances in understanding glioblastoma biology and promising results of several preclinical studies, new therapies for glioblastoma have failed to improve patient survival^7^.

There are several reasons why treating glioblastoma is so difficult. Its infiltrative nature and fragility of surrounding brain tissues make complete resection of tumor cells nearly impossible, leading to tumor recurrence at the resection margin^2,8^. Another is that the presence of mutations in genes such as epidermal growth factor receptor (EGFR), isocitrate dehydrogenase (IDH), protein kinase C (PKC), tumor protein p53 (p53), phosphatase and tensin homolog (PTEN), retinoblastoma protein (pRB), and vascular endothelial growth factor (VEGF) leads to the deregulation of major cellular pathways, ultimately resulting in high proliferative activity of GBM cells and the promotion of continuous neovascularization in GBM tumors^7–10^. Moreover, the blood-brain barrier limits the penetration of chemotherapeutics to the tumor area^7,8^. Treatment is further impaired by substantial heterogeneity of the tumor and the presence of a population of chemo- and radiotherapy-resistant cancer-stem-like cells (CSCs)^8,11^. Finally, GBM cells secrete numerous soluble factors that reshape the tumor microenvironment, enhancing cancer cell proliferation and impairing antitumor immune response^7,8,12^. Some studies have proposed midkine (MDK), a soluble heparin-binding growth factor, as one of them^13–16^.

In a healthy central nervous system (CNS), MDK is produced by fetal astrocytes during neurogenesis^17,18^. Then, its expression is progressively decreasing to low levels in adults^19,20^. However, MDK can be conditionally expressed in adults upon tissue damage due to its involvement in regenerative processes inducing cell proliferation, monocyte chemotaxis, and chemokine expression^13^. MDK signals through multiple receptors, including proteoglycans such as syndecans and receptor protein tyrosine phosphatase-β (RPTP-β), as well as non-proteoglycan receptors such as anaplastic lymphoma kinase (ALK), low-density lipoprotein receptor-related protein-1 (Lrp1) and integrins (α4β1 and α6β1)^21–26^. Its multifaceted properties, normally utilized during embryogenesis or tissue regeneration, can be hijacked in cancer^13^. MDK is overexpressed and has diagnostic and prognostic potential in several cancers, including lung, bladder, esophageal, and breast cancer^27^. MDK pleiotropic downstream signaling events transduced through its vast array of receptors are involved in multiple hallmarks of cancer, including proliferative signaling via direct activation of its receptors, inducing neovascularization, avoiding immune destruction via a chemotactic effect on macrophages, which are then being M2 polarized, and direct effect on promoting invasion and metastasis, as reviewed in detail by Filippou et al.^13,27,28^.

In gliomas, studies of midkine (MDK) started in 1997 when Mishima et al. showed that MDK mRNA and protein expression levels were higher in high-grade astrocytomas, e.g., anaplastic astrocytomas and glioblastomas, compared to low-grade astrocytomas and healthy brain^29^. Further studies indicated that high MDK expression is associated with a worse prognosis in GBM patients^30^. MDK was also shown to increase GBM cell proliferation, migration, and invasion and induce temozolomide resistance by improving the stem-like properties of GBM through ALK and Notch homolog 1 (Notch1)/phosphorylated c-Jun N-terminal kinase (p-JNK) signaling pathways^16,22,31^. Additionally, a recent study revealed that MDK rewires tumor-infiltrating T cells to secrete chemokines contributing to protumorigenic microenvironment^32^. The mechanism of its overexpression in glioma has not been elucidated. However, it’s been suggested that retinoic acid, Wnt/β-catenin, and hypoxia signaling play a role^33–35^. Despite evidence of its importance in GBM, large-scale studies confirming MDK’s expression patterns and cellular source are lacking. Moreover, many of its biological functions in glioblastoma remain poorly understood.

In view of its multifaceted role and insights from recent preclinical and Phase I clinical studies, we rationalized performing a comprehensive characterization of *MDK* expression patterns together with MDK-associated multiomic footprint in glioblastoma to lay the ground for future investigations and allow for informed design of clinical trials based on identifying patients that will likely benefit from the treatment. We collected primary tumor samples, established matched primary GBM cell cultures, and performed the transcriptomics and proteomics using RNA sequencing, enzyme-linked immunosorbent assay (ELISA), and Luminex. We then harnessed the power of multiple glioma datasets: The Cancer Genome Atlas (TCGA)-GBM, TCGA-LGG, Chinese Glioma Genome Atlas (CGGA), ERP010930, and SRP044668 to create a large, harmonized RNA-seq dataset with 1017 gliomas and healthy brain tissue samples. Single-cell RNA-seq, methylation, and mutational analysis of primary glioblastoma tumors from the TCGA-GBM cohort further complemented the study. For additional validation, we stimulated macrophages with either MDK or GBM cell culture supernatants to confirm MDKs’ impact on macrophage secretome.

Overall, our study confirmed earlier findings of increased MDK expression in gliomas, proportional to the tumor grade, as well as its association with poor survival in GBM with a diverse 1000 + patient cohort. Additionally, we have discovered a dominant MDK isoform with evidence for selection pressure towards its expression along with an increase in tumor grade. Finally, our multiomic analyses revealed an unfavorable MDK-associated footprint and its role in reshaping the TME by inducing an immunosuppressive secretome of macrophages. Altogether, this underscores the importance of MDK research in developing targeted therapies for GBM.

## Materials and methods

### Human samples

The study was conducted according to the principles of the Declaration of Helsinki and local regulations. Biological material from patients with glioblastoma was obtained and processed as described in Gozdz et al.^36^. Briefly, glioblastoma (GBM) tissue and plasma samples were collected from patients undergoing surgery at the Medical University of Warsaw Neurosurgery Clinic and the Regional Hospital of the Medical University of Silesia in Sosnowiec. The tissue samples were used to establish primary GBM cell cultures and for bulk RNA-seq, while the plasma was used for proteomics, as described below.

The study was approved by the Bioethics Committee of the Medical University of Silesia (permit number KNW/0022/KB1/2/I/17). All patients participating in the study provided informed consent before the surgery. Shortly before surgical resection, venous blood samples were collected on K2 EDTA from 9 patients (7 males, 2 females; median age 59 years, min. 41, max. 71). Plasma was then obtained by centrifugation (2000 × *g* for 15 min), after which it was partitioned and frozen at −80 °C until further analysis. All tumors were classified histopathologically as glioblastoma. For RNA-Seq analysis, we used tumor samples from 12 donors (8 males, 4 females; median age 61 years, min. 41, max. 71), and 12 ex-vivo cultures established from these tumors.

Tumor tissue was processed one to six hours after resection. A portion of the tissue was quick frozen for later nucleic acids isolation, and the rest was washed twice with PBS containing 5% BSA and antibiotics, then cut into small fragments with a scalpel with blade no.10 until it was well minced (adopted from Leelatian et al.^37^. The tissue was then transferred to 15 mL conical tubes by flushing the dish with the 5 mL activated dissociation medium (containing collagenase IV and DNAase I) and incubated at 37 °C for 60 min. After this time, the tube was centrifuged for 5 min at 300 × *g* and resuspended in ACK lysis buffer for 10 min. After another centrifugation and passing through a 70 μm cell strainer and then a 40 μm cell strainer, the cells were resuspended in a tumor sphere culture medium. The medium was composed of DMEM-F12 with Glutamax (#31331028, Gibco), 2% B27 supplement (#0080085SA, Gibco), 20 ng/mL EGF (#PHG0311, Gibco), 20 ng/mL bFGF (#PHG0026, Gibco), 0,5% Penicilin-Streptomycin solution (#15140122, Gibco), and 1 IU/mL heparin (#7980, STEMCELL Technologies). The culture was started by seeding 2 × 10^6^ cells in 10 mL of tumor sphere culture medium in a T25cm^2^ culture flask. Immediately upon reaching an exponential growth rate, cells were propagated for cryopreservation, and the day of the first cryopreservation was acknowledged as the date of cell line establishment.

### Maintenance of spherical ex vivo cultures derived from glioblastoma and culture of commercially available GBM cell lines

The primary ex vivo glioblastoma spherical cell cultures were maintained in tumor sphere culture medium composed of DMEM/F12 with Glutamax, 2% B27 supplement, 20 ng/ml EGF, 20 ng/ml bFGF (all Gibco, Thermo Fischer Scientific), 0.5% Penicillin-Streptomycin solution (Gibco) and 1 IU/ml heparin (STEMCELL Technologies) in an incubator under standard culture conditions (37 °C, 5% CO^2^). Cells were grown as non-adherent, three-dimensional spheroids on 25 cm^2^ cell culture flasks (Sarstaedt), or (2 populations which from the beginning showed a tendency to adhere to surfaces) in an adherent form on laminin-coated 25 cm^2^ culture flasks (Sigma Aldrich). The culture medium was replaced twice a week by centrifugation (5 min at 350 × g). Spheroids were passaged by accutase digestion (ACCUMAX, Sigma Aldrich) every 14 days. Cells between 7 and 14 passages were used for analyses.

To analyze the effect of conditioned medium from GBM culture on the secretion profile of macrophages, cells from the 9 GBM populations were seeded at a density of 3 million cells per well in a 6-well plate in standard GBM medium in three replicates and cultured for 2 days until spheroids were formed. The medium was then replaced, and the cells were cultured in 2 ml of medium per well for 72 h, after which the medium was collected, pooled from 3 replicates into a single sample, aliquoted, and frozen at −80 °C for further use. At the time of medium collection, there were 5–8 million GBM cells in each well, depending on the population.

The U-251 cell line was purchased from Sigma-Aldrich (Merck), the DKMG line from DSMZ (GmbH) and the U87 line from ATCC. The lines s were cultured in MEM Complete Medium, with non-essential amino acids, 1 mM sodium pyruvate, 2 mM L-Glutamine and 10% FBS and 1% penicillin-streptomycin (all Gibco, Thermo Fischer Scientific). Cells were maintained at 37 °C in a humidified atmosphere and 5% CO2 until 80% confluency was achieved and then detached with TrypLE™ Express (Thermo Fischer Scientific).

### Analysis of the secretory profile of macrophages

The effect of midkine (MDK) and conditioned media from GBM spherical cultures on the secretion profile of macrophages was investigated using cells obtained from buffy coats from three healthy donors purchased from the Regional Blood Donation and Blood Treatment Centre in Warsaw, in accordance with the consent of the Bioethics Committee at the Medical University of Warsaw No. AKBE/20/2022. PBMC isolation was performed using Histopaque^®^−1077 (Merck) with SepMate™ PBMC Isolation Tubes (STEMCELL Technologies) according to the manufacturer’s protocol. The PBMC were then seeded into 48-well plates for non-adherent culture (Sarsteadt) at 550,000 cells per well (two complete plates from each donor) in RPMI (Gibco) with 10% human serum (Merck). After two hours in the incubator, the plates were removed, the medium was aspirated, and all wells were thoroughly rinsed twice with DPBS (Gibco). The cells adhering to the bottom were cultured for another 4 days in RPMI with 10% human serum. After four days, the cells reached the macrophage phenotype, which was confirmed by flow cytometry, and then treatment with midkine and conditioned medium from GBM culture was initiated. After removing the existing medium, 200 µl of RPMI medium with 10% human serum enriched with 4 concentrations of MDK (#450-16-20UG, Peprotech) were added to the wells: 100 ng, 500 ng, 1 µg, and 2.5 µg. The control cells for these treatments were those cultured in 200 µl RPMI with 10% human serum. To the cells treated with conditioned medium from the culture of 9 primary GBM populations, 200 µl of medium consisting of a 1:1 mixture of conditioned GBM medium (DMEM/F12 + 2% B27 Supplement + 20ng/ml bFGF + 20ng/ml EGF + 1IU/ml heparin, collected after 72 h of spherical culture) and RPMI with 10% human serum were added. To the cells treated with midkine neutralizing antibody (#MA5-32538, Invitrogen, Thermo Scientific) before adding 200 µl of the mixture of conditioned GBM medium with RPMI to the wells with macrophages, neutralizing antibodies were added to the conditioned medium and incubated for 15 min, then mixed with the appropriate RPMI medium and applied to the wells. The final concentration of neutralizing antibody in the GBM and RPMI media mixture was 15 µg/ml. The control cells for this treatment were those treated with 200 µl of a mixture of pure GBM medium and RPMI 10% human serum in a 1:1 ratio. Each treatment was performed in 3 technical replicates (3 wells per treatment) for each of the 3 donors. The treatments were conducted for another 72 h, after which the medium was collected, pooled from 3 technical replicates into one sample, centrifuged at 800 x *g* for 4 min, and frozen in Eppendorf tubes at −80 °C for Luminex analysis.

### Western blotting

To confirm intracellular midkine expression at the protein level by Western blot analysis, spherical cultures of 10 GBM populations were maintained under standard GBM culture conditions (T25cm^2^ bottles, DMEM/F12 medium with 2% B27 Supplement, 20ng/ml EGF, 20ng/ml bFGF and 1IU/ml heparin, at 5% CO2 and 37 °C) for at least three consecutive weeks until approximately 6 million cells were obtained from each bottle. The cultures were then centrifuged (350 x *g*, 5 min), the media were discarded, and the spheroids were digested with StemPro Accutase Cell Dissociation Reagent (#A1110501, Gibco, Thermo Scientific). The cells were then washed twice in DPBS (Gibco), and after decanting, the cell pellet was frozen at −80°C. Protein isolation was performed by suspending the cell pellets in 100 µl of RIPA Lysis and Extraction Buffer (Thermo Scientific) with the addition of protease and phosphatase inhibitors (Cell Signalling, #5871 and #5870, respectively) for 30 min on ice. The cell lysates were then centrifuged (14,000 rpm for 20 min at 4 °C), transferred to new Eppendorf tubes, and subjected to protein quantification using the Pierce BCA Protein Assay Kit (#23225, Thermo Scientific). After isolation and determination of protein levels, the lysates were frozen at −80°C. For WB analysis, 4xLaemmli Sample buffer (#1610747, BioRad) with 355 mM β-mercaptoethanol was added to samples containing 15 µg of total protein and denatured at 100 °C for 10 min before loading onto the gel. Electrophoretic separation of proteins was performed on a 12% polyacrylamide gel (29:1) in TRIS-HCL, Glycine, SDS buffer at a constant voltage of 180 V using PageRuler™ Plus Prestained Protein Ladder (Thermo Scientific) protein marker, applying 15 µg of protein lysate per well. Next, the proteins were electrotransferred to a methanol-activated Immobilon-FL PVDF Membrane (Millipore, Merck) in TRIS-HCL, Glycine, 20% methanol buffer for 110 min at a constant voltage of 100V. The membranes were then washed, and the protein bands were detected by staining with Ponceau S red and imaged with the ChemiDoc MP System (Bio-Rad) using the colorimetric detection method. The membranes were then rinsed in water until the red dye was thoroughly washed out and blocked for 1 hour in a 5% solution of non-fat milk powder in TBST. The blocked membranes were then incubated overnight at 4 °C in a solution of primary antibodies. The next day, the membranes were washed in TBST (4 × 10 min) and incubated in a solution of secondary antibodies conjugated with HRP for 1 hour at room temperature. After another cycle of washing in TBST, the membranes were developed using Clarity Max ECL (BioRad) for 5 min and imaged on a ChemiDoc MP Imaging System (Bio-Rad) with the ‘Blots’ and ‘Chemiluminescence’ application. Densitometric data were acquired using Image Lab software (Bio-Rad, v.6.1.0 build 7 Standard Edition for Windows, Bio-Rad Laboratories Inc., Hercules, California, USA, www.bio-rad.com/en-pl/product/image-lab-software? ID=KRE6P5E8Z) with data analysis, graphing and statistics performed using GraphPad Prism software (v.10.5.0 for Windows GraphPad Software, Boston, Massachusetts USA, www.graphpad.com). The list of utilized antibodies can be found in Supplementary Table 1.

### Luminex and ELISA

Quantitative assessment of MDK secretion in patient plasma and media from primary ex-vivo cultures and GBM cell lines culture was performed on undiluted samples by Luminex’s technology on a LUMINEX 200 platform (Luminex Corporation) using the Human Luminex Discovery Assay kit (#LXSAHM, R&D Systems) according to the manufacturer’s protocols. For this purpose, cells from 12 GBM populations (approximately 3 million per population) were cultured for 48 h in 5 mL of tumor sphere culture medium, under standard conditions (37 °C, 5% CO^2^), on 25 cm^2^ flasks. The culture medium was then collected and centrifuged (350 × *g* for 5 min), divided into aliquots, and frozen at −80 °C for further analysis. The culture medium of the U251, DKMG, and U87 cell lines for secretion assays was collected after 48 h of cell culture in 2 ml of complete MEM medium in 6-well plates after centrifugation of 450 × *g*. On the day of analysis, the collected medium was thawed on ice. The assay procedure was performed according to the manufacturer’s protocol. The results were normalized by the values obtained in the basal growth medium and the number of cells collected from the culture flask. As the results of midkine concentrations in GBM patients’ plasma and primary cultures were very high, we decided to verify the analysis with the Human Midkine ELISA kit (Abcam, #Ab193761) according to the manufacturer’s protocol on 5-fold diluted samples.

A Luminex analysis evaluating the effect of midkine and conditioned media from primary GBM cultures on the secretory profile of macrophages was performed using the Human Luminex Discovery Assay kit (#LXSAHM, BioTechne) with the following analytes: CCL2, MIP-1α (CCL3), CCL8, CCL13, CCL14, CCL17, CCL18, CCL26, CXCL5, CXCL6, CXCL10, CXCL11, interleukin 1 alpha (IL-1α), interleukin 10 (IL-10), interleukin 15 (IL-15), interleukin 33 (IL-33), interferon γ (IFN-γ), granulocyte-macrophage colony-stimulating factor (GM-CSF), granzyme B, programmed death-ligand 1 (PD-L1), and platelet-derived growth factor AA/BB (PDGF-AA/BB), selected based on transcriptomic data. On the day of analysis, frozen portions of macrophage culture medium and control media were thawed on ice and applied to the plate without diluting. The analysis was performed according to the manufacturer’s protocol. The results of the analysis were processed using Belysa Immunoassay Curve Fitting Software (v.1.2 for Windows, Cat. No. 40–122, Merck KGaA, Darmstadt, Germany https://www.sigmaaldrich.com/PL/pl/product/mm/40122?srsltid=AfmBOorlbA0a8uh9IhRwaGOPL0JvCiPBc5kNGdiHuWGCs-53IAgFYnFQ).

### Isolation of RNA from tissues and cell cultures and RNA sequencing

Cryopreserved tumor sections and cells cultured at 20% and 5% oxygen were harvested at the same or adjacent passage, mechanically disrupted in TRIzol Reagent (Thermo Fisher Scientific), and processed along the manufacturer’s instructions to obtain material for RNA sequencing. The integrity of the isolated RNA was assessed using a Bioanalyzer (Agilent Technologies). RNA sequencing was performed on high-quality RNA samples isolated from 12 GBM tumors and 12 tumor-derived ex-vivo cultures. Sequencing was performed on a HiSeq 1500 (Illumina) in a rapid run flow cell with paired-end settings (2 × 76 bp). RNA sequencing reads were processed using nf-core/rnaseq; they were aligned to the human genome (GRCh38) using the HISAT2 (v2.2.0) algorithm, then transcripts were assembled and quantified using StringTie (v2.1.7)^38,39^.

### Data mining and preprocessing

All analyses were performed in R (https://www.r-project.org/) using Bioconductor (https://www.bioconductor.org/) packages. Tidyverse R package was used for data preparation. ggplot2, Nebulosa, and ComplexHeatmap R packages were used for data visualization^40–42^.

The bulk gene expression profiles (raw counts from RNA-seq) of four glioma datasets as well as healthy brain tissues were downloaded from recount-brain, a curated repository for human brain RNA-Seq datasets, with cohort names: TCGA-GBM, TCGA-LGG, Chinese Glioma Genome Atlas (CGGA), ERP010930, and SRP044668^9–11,43–46^. All available metadata, including age, grade, race, sec, *IDH* mutation status, and overall survival data, were downloaded from recount-brain, the Cancer Genome Atlas (TCGA) and CGGA repositories. Isocitrate dehydrogenase (*IDH*) mutation status in SRP044668 was kindly provided by Dr. Peter A. Sims. Data from http://www.brainrnaseq.org was kindly provided by Dr. Steven Sloan^47,48^. Retrieving data from the recount ensured that consistent bioinformatic pipelines were used for these four datasets^43^. To further ensure no batch effects between the datasets, we performed batch effect removal by the ComBat_seq method from the R package “sva” (v3.48) to remove unwanted variation in our combined dataset. Tumor grade was included as a covariate^49^.

The transcript isoform percentage of glioblastoma, low-grade glioma, and healthy brain tissues were downloaded from UCSC Xena (https://xenabrowser.net/datapages/) - cohort TCGA TARGET GTEx filtered for gliomas (grade II-IV), and healthy brain tissues, accession date 25th July 2022.

The single-cell gene expression profiles of glioblastoma from 28 patients (24131 cells) were downloaded from Single Cell Portal, accession date 26th October 2021 (SPC393, https://singlecell.broadinstitute.org/single_cell)^50^.

### Group assignment and survival analysis

Patients in the combined RNA-seq dataset were dichotomized for survival analysis into two groups based on MDK expression. The optimal cutoff point was determined using maximally selected rank statistics, defined as the expression threshold that yields the most significant split based on the standardized log-rank test, implemented via the maxstat and survminer (v0.4.9) R packages. Based on this cutoff, patients were assigned to either the *MDK*^high^ or *MDK*^low^ group. The dataset was then stratified by tumor grade or by both grade and *IDH* mutation status. Kaplan–Meier survival analysis with log-rank testing was performed to assess differences in overall survival between the two groups.

A global cutoff was used to dichotomize samples for survival analysis across all glioma grades and *IDH* statuses, ensuring consistent stratification and enabling direct comparison of MDK’s prognostic value across glioma subtypes. For downstream multiomic analyses focused on grade IV *IDH*^wildtype^ gliomas—where MDK showed significant prognostic relevance—a subgroup-specific cutoff calculated in an identical manner was used to better capture biologically meaningful differences within this cohort.

### Differential gene expression analysis

For differential gene expression analysis, the RNA-seq expression data was either raw count type (combined bulk RNA-seq dataset) or imported from StringTie (our dataset)^38^. Patients were divided into two groups based on either MDK expression as described above (recount v2 datasets) or divided based on sample type (primary tumor vs. primary cell culture). After assigning the samples to two groups based on risk score, we performed differential gene expression analysis using *DESeq2* R package^51^. To optimize power, we used Independent Hypothesis Weighting (IHW) with an adjusted p-value threshold < 0.05 to report differentially expressed genes (DEGs) and further gene-set enrichment analysis^52^. Gene set enrichment analysis was performed using *fgsea* R package (v1.26)^53^.

### Estimation of immune cell type abundance in tumor tissue

Patients in the combined RNA-seq dataset were divided into two groups based on either MDK expression as described above. We used *CIBERSORTx* (https://cibersortx.stanford.edu/) to evaluate the relative abundance of predefined cell types in mixed solid tissues^54^. Normalized and harmonized TPM gene expression data was used for this analysis. We employed the LM22 leukocyte gene signature matrix downloaded from the *CIBERSORTx* website. LM22 contains 547 genes distinguishing 22 types of immune-related cells. We set the number of permutations to 1000 for robust analysis. The B-mode of batch correction supplied with LM22 GEP was used. *CIBERSORTx* enumerated the abundance scores of the 22 infiltrating immune cells, including B cells, dendritic cells, T cells, natural killer cells, macrophages, and others. To simplify the text, we referred to the cell type abundance estimates as “abundance” or “infiltration” alone. The results were filtered with a *p-value* < 0.05 threshold.

### Methylation and mutation analysis

Patients in the combined RNA-seq dataset were divided into two groups based on either MDK expression as described above. The methylation data for the TCGA-GBM cohort was obtained using *ELMER* (v2.24.0, accession date 8th October 2024) and processed as described in Silva et al.^55,56^. The mutation data for the TCGA-GBM cohort was obtained using *maftools* (v2.16.0, accession date 16th June 2022) and processed as described in Silva et al.^55,56^.

### Single-cell RNA sequencing analysis

Data was accessed as described above and imported into Seurat (v4.1.0) along with meta- and clustering data^57^. Malignant cells were split based on MDK expression (positive or negative). Differential gene expression analysis was performed using FindMarkers (Seurat). Gene set enrichment analysis was performed using fgsea (v1.26)^53^. We utilized cell cycle markers from Kowalczyk et al. 2015^58^, to assign cell cycle state to each malignant cell. Cell cycle analysis was performed using CellCycleScoring implemented in Seurat.

## Results

### Midkine expression in healthy brain tissues and gliomas

To characterize and better understand the role of midkine (MDK) in gliomas, we downloaded uniformly processed RNA-Seq abundance values from recount-brain, a curated repository for human brain RNA-Seq datasets, for four different uniformly processed datasets of adult gliomas and healthy brain tissue controls: 643 glioma samples from the cancer genome atlas (TCGA), 268 glioma samples from Chinese glioma genome atlas (CGGA), 93 glioma samples from SRP044668 and 48 glioma samples from ERP010930. For expression analysis, 35 samples from the resection margin were excluded. For the survival analyses, we included only patients with complete survival data. Retrieving data from the recount ensured that consistent bioinformatic pipelines were used for these four datasets. To further ensure no batch effects between the datasets, we performed batch effect removal using negative binomial regression. Then we retrieved all available metadata and used isocitrate dehydrogenase (*IDH*) mutation status and tumor grade to align our cohort to the 2021 World Health Organization (WHO) Classification of Gliomas, which groups adult-type diffuse astrocytic tumors based on *IDH* mutation status, with only *IDH*^wildtype^ tumors with either characteristic morphology or molecular features of glioblastoma being classified as "glioblastoma, *IDH*^wildtype^"^3^.

**Fig. 1:**
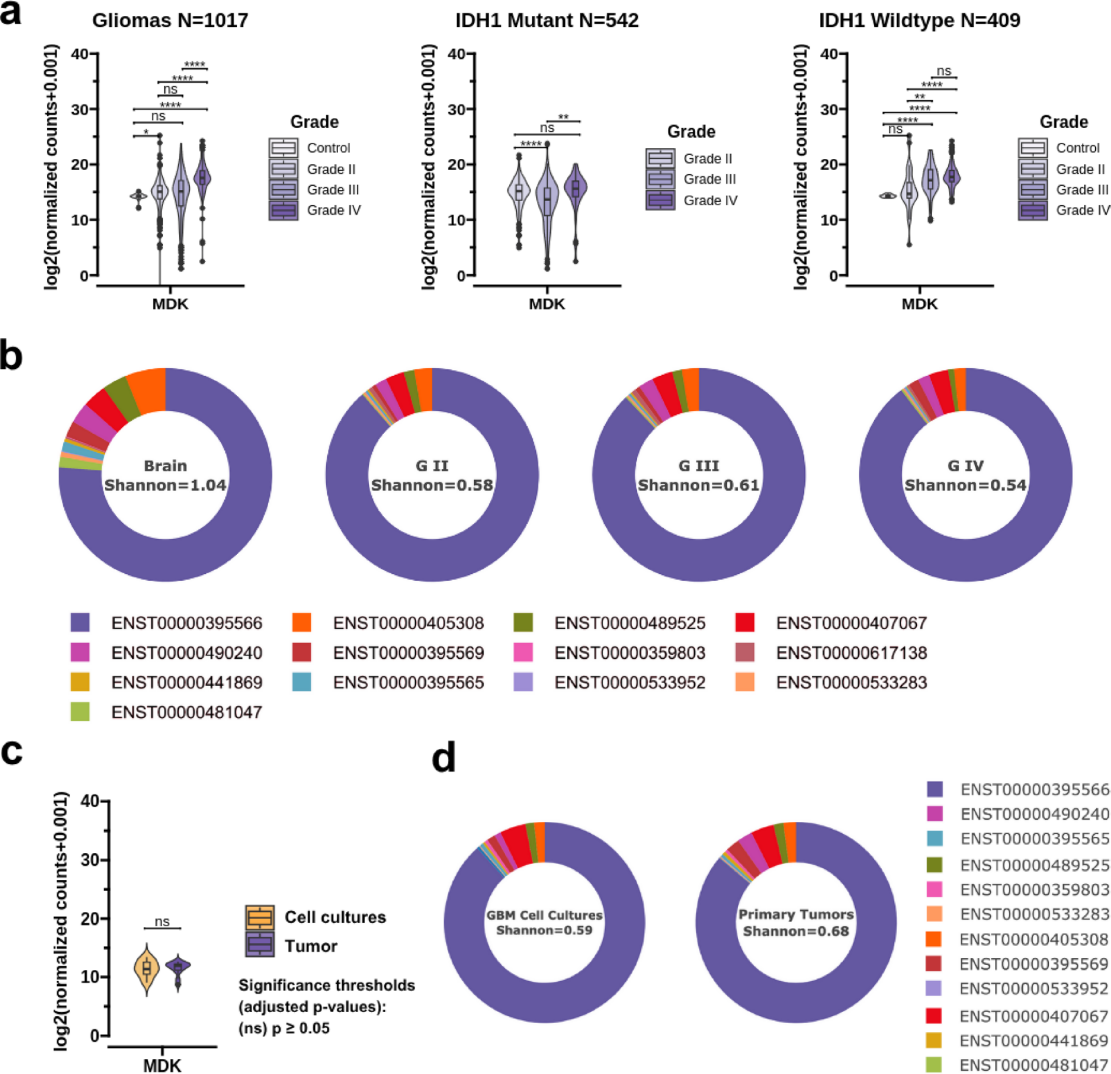
Midkine expression in healthy brain tissues and glial tumors. (**a**) Midkine expression (log2(DESeq2 normalized counts + 0.001) in the combined dataset in all gliomas and healthy brain tissues stratified by grade (*N* = 1017) (left), in *IDH*^mutant^ gliomas (*N* = 542) (center), and *IDH*^wildtype^ gliomas and healthy brain tissues stratified by grade (*N* = 409). (**b**) *MDK* isoform proportions in grade II, III, and IV gliomas and healthy brain tissues from TCGA (672 samples) and GTEX (1146 samples) datasets. Shannon diversity index was calculated for each group. (**c**) Midkine expression (log2(DESeq2 normalized counts + 0.001) in the tumor tissues (*N* = 12) and the primary GBM cell cultures (*N* = 12) in the WUM cohort. (**d**) *MDK* isoform proportions in the tumor tissues (*N* = 12) and the primary GBM cell cultures (*N* = 12) in the WUM cohort. Wilcoxon rank-sum test was applied to evaluate the differences between sample types in (**a**) and (**c**). Significance thresholds: NS p > = 0.05; ** *p* < 0.01; *** *p* < 0.001; **** *p* < 0.0001.

Hijacking cytokine-signaling is a hallmark of cancer, and understanding the role of these hijacked cytokines in tumor samples can help researchers design novel treatments. Therefore, we evaluated *MDK* expression in healthy brain tissues and gliomas in our combined dataset. At first, our analysis revealed a consistent picture of high *MDK* expression in grade II, III, and IV gliomas compared to healthy brain tissues, with its expression increasing proportionally to the tumor grade (Fig. 1a). Stratifying the dataset based on *IDH* mutation status revealed that MDK expression is low and relatively stable across different tumor grades in *IDH*^mutant^ tumors (Fig. 1a). Conversely, it is higher and increases proportionally to the tumor grade in *IDH*^wildtype^ tumors (Fig. 1a, Supplementary Fig. 1). Overall, MDK expression was higher in *IDH*^wildtype^ grade III and IV tumors than *IDH*^mutant^ tumors of corresponding grade (Supplementary Fig. 1).

Healthy brain tissues were characterized by scarce *MDK* expression, with the fetal astrocytes being the only central nervous system (CNS) cell type expressing *MDK* under physiologic conditions (Supplementary Fig. 2). mRNA isoform analysis revealed that the most prevalent *MDK* isoform remained the same (ENST00000395566) in all sample types. However, along with the tumor grade, the prevalence of ENST00000395566 increased, while the diversity of *MDK* isoforms gradually decreased, as assessed by the Shannon diversity index (Fig. 1b).

Subsequently, we collected 12 primary glioblastoma (GBM) tumors and derived 12 primary tumor ex vivo cultures. We then performed RNA sequencing on both tumor tissues and matched primary cultures. *MDK* expression was comparable in the tumor samples and respective primary cell cultures, suggesting that malignant cells are the predominant MDK source in GBM tumor microenvironment (TME) (Fig. 1c). Notably, the mean *MDK* isoform proportions were similar to gliomas in the combined dataset (Fig. 1d). The primary tumor samples had greater *MDK* isoform diversity compared to primary tumor ex vivo cultures (Fig. 1d).

### Profile of midkine expression in glioblastoma

**Fig. 2:**
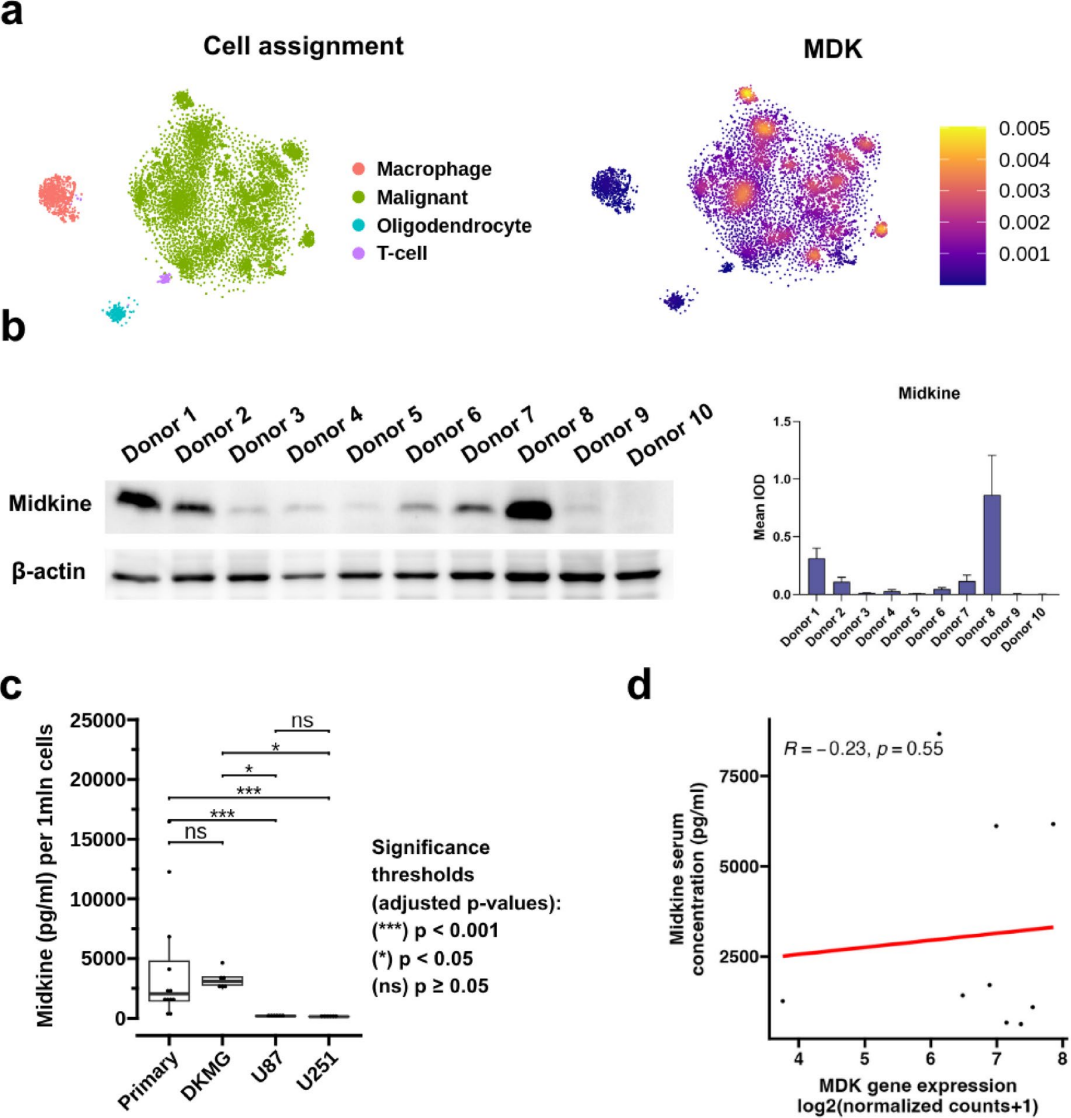
Cross-section of MDK in different samples. (**a**) Midkine expression on a single-cell level from primary glioblastoma tumors (*N* = 28) projected on tSNE plot (*N* = 7930 cells). (**b**) Midkine protein expression in lysates from primary glioblastoma cell cultures from the WUM cohort (*N* = 10) assessed by Western blot. Representative image of the chemiluminescent signal of midkine bands followed by membrane stripping and probing for b-actin. The mean integrated optical density (IOD) from three experiments is presented as a barplot on the right side of the panel (*N* = 10). Error bars represent the SEM from the average of three separate experiments. (**c**) Midkine secretion by primary glioblastoma cell cultures (*N* = 12) and DKMG, U87, and U251 cell lines. Secretion was evaluated on cell culture medium using Luminex. Wilcoxon rank-sum test was applied to assess the differences between cell types. (**d**) Correlation of MDK serum concentration (pg/ml, y-axis) with *MDK* mRNA expression log2(normalized counts + 1) on the x-axis (*N* = 9). Spearman’s rank correlation was used to assess the relationship between variables.

In search for other cell types potentially contributing to the MDK pool, we analyzed single-cell RNA-seq dataset from 28 GBM patients (7930 cells) that included myeloid cells, oligodendrocytes, and T cells along with malignant cells (Fig. 2a). The expression data indicates that malignant cells, indeed, are the predominant source of *MDK* in the tumor microenvironment. To further validate these findings, we performed western blotting of ten GBM primary cell cultures, which confirmed expression of MDK in GBM cells (Fig. 2b, Supplementary Figs. 3 and 4), as well as western blotting of macrophages and macrophages stimulated with GBM-conditioned media, both with no signs of MDK expression (Supplementary Fig. 4). To investigate potential mechanisms leading to high MDK expression, we examined the correlation of MDK with retinol binding protein 1 (*RBP1*) expression as well as with HALLMARK_HYPOXIA and HALLMARK_ESTROGEN_RESPONSE_LATE signature scores (Supplementary Fig. 5a-c). The correlation coefficients were *R* = 0.59, *R* = 0.32, and *R* = 0.28, respectively (*p* < 2.2e-16 for all).

### Midkine secretion by malignant cells

After confirming malignant GBM cells as the primary source of *MDK* mRNA expression, we proceeded to evaluate MDK secretion to determine whether its expression is reflected at the secretion level. For this purpose, we performed Luminex and Enzyme-Linked Immunosorbent Assay (ELISA) assays on supernatants from 12 primary cell cultures and three commercially available GBM cell lines: DKMG, U251, and U87. MDK was secreted by all the primary cell cultures, reaching a median concentration of 2049 pg/ml (per 1 million cells) after a 48-hour culture in 5 ml (Fig. 2c). Among commercially available cell lines, DKMG was the only one to secrete significant quantities of MDK (median concentration of 3244 pg/ml per 1 million cells), whereas U87 and U251 only secreted negligible amounts (140pg/ml and 178pg/ml per 1 million cells, respectively).

With the substantial potential of primary glioblastoma cells for MDK secretion, we decided to assess whether local expression correlates to a systemic rise of MDK levels, which could potentially be used for the non-invasive identification of *MDK*^high^ tumors. For this purpose, we analyzed plasma samples from 9 GBM patients with matched tumor bulk RNA-seq data. While midkine serum concentration was higher than the norms reported for healthy people, there was no correlation between MDK expression and MDK serum concentration (*R* = −0.23, *p* = 0.55) (Fig. 2d).

### Prognostic value of midkine in glioblastoma

**Fig. 3:**
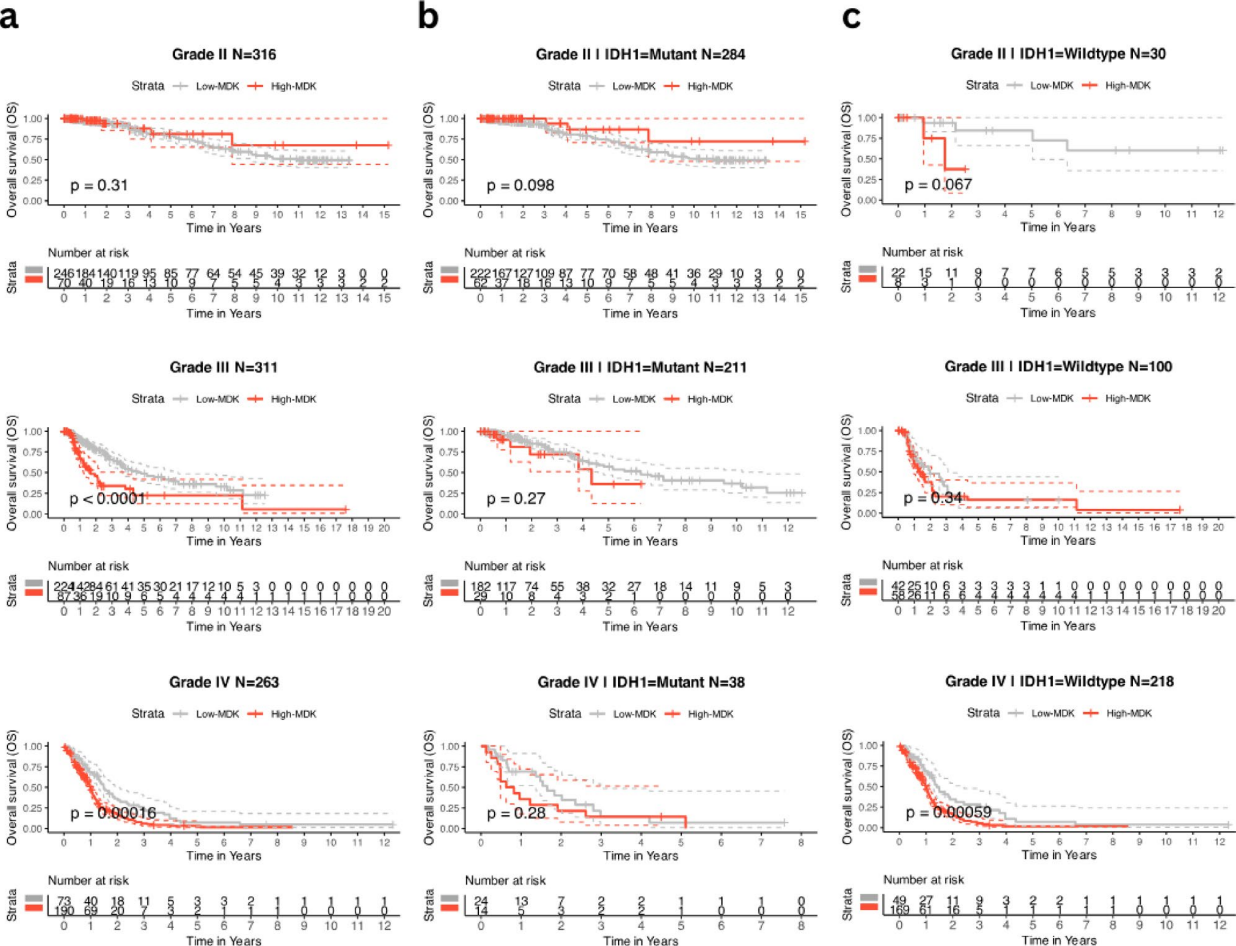
Prognostic role of *MDK* in gliomas. Kaplan–Meier survival analysis of overall survival based on MDK expression in combined glioma dataset. In (**a**) the combined dataset is stratified by grade only into Grade II (N = 316), Grade III (N = 311), and Grade IV (N = 263) subgroups. In (**b**) *IDH*^mutant^ tumors are stratified by grade into Grade II *IDH*^mutant^ (N = 284), Grade III *IDH*^mutant^ (N = 211), and Grade IV *IDH*^mutant^ (N = 38). In (**c**) *IDH*^wildtype^ tumors are stratified by grade into Grade II *IDH*^wildtype^ (N = 30), Grade III *IDH*^wildtype^ (N = 100), and Grade IV *IDH*^wildtype^ (N = 218). Below each plot is a ‘number at risk’ table - the cumulative number of events table and the cumulative number of censored subjects table. Statistical comparison was performed by using the log-rank test.

The existing selective pressure for higher *MDK* expression, favoring its specific isoform (ENST00000395566), suggests its tumor-promoting role in gliomas. To assess its prognostic significance, we have performed Kaplan-Meier univariate Cox regression analyses in gliomas using *MDK* expression as an overall survival (OS) predictor. First, patients were split into *MDK*^high^ and *MDK*^low^ groups based on the abundance of *MDK* using the maxstat algorithm, as described in detail in materials and methods. Then, the tumors were stratified by either grade (Fig. 3a) or grade and *IDH* mutation status (Fig. 3b & c). Both Kaplan-Meier and Cox regression survival analyses showed that patients with *MDK*^high^ tumors had significantly shorter OS than those with *MDK*^low^ tumors in Grade III and Grade IV gliomas (both *IDH*^wildtype^ and *IDH*^mutant^ tumors combined) (Fig. 3a, Supplementary Fig. 6a), as well as *IDH*^wildtype^ gliomas in general (Grades II, III and IV) (Supplementary Fig. 6b and Supplementary Fig. 7a-b). Further analysis stratified by both grade and *IDH* mutation status revealed that only in grade IV *IDH*^wildtype^ tumors high *MDK* expression was significantly associated with poor prognosis (HR = 1.94, CI = 1.32–2.85, *p* < 0.001) (Fig. 3b-c and Supplementary Fig. 6c-d).

### Single-cell map of *MDK*^positive^ and *MDK*^negative^ glioblastoma cells

**Fig. 4:**
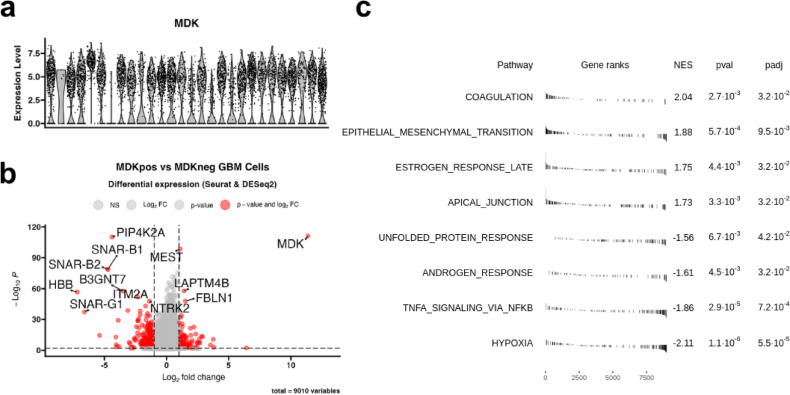
Comparison of *MDK*^positive^ and *MDK*^negative^ malignant cells. (**a**) MDK expression at single-cell level in glioblastoma tumor tissue (*N* = 28 donors and 7930 cells). (**b**) Differential gene expression analysis between *MDK*^positive^ (*N* = 5041 cells) and *MDK*^negative^ (*N* = 1822 cells) malignant cells (**c**) Gene-set enrichment analysis (Hallmark gene set) of differentially expressed genes between *MDK*^positive^ (*N* = 5041 cells) and *MDK*^negative^ (*N* = 1822 cells) cells. Significantly enriched pathways are shown, with normalized enrichment scores and adjusted p-values.

After confirming MDK’s role in GBM prognosis, we explored its biological effects through multiomic analysis. First, we investigated the *MDK* expression in GBM cells at single-cell level in single cell RNA-seq data. The *MDK* expression was heterogeneous in most donors, however, the vast majority of malignant cells were *MDK* positive (Fig. 4a). That prompted us to explore the differences between *MDK*^negative^ and *MDK*^positive^ cells through differential gene expression analysis followed by pathway enrichment analysis (Fig. 4b).

Some of the most downregulated genes in *MDK*^positive^ cells were *HBB* (hemoglobin beta chain), *ITM2A* (Integral Membrane Protein 2 A), and *LOX* (Lysyl Oxidase) (Fig. 3b and Supplementary File 1). Conversely, *MDK*^positive^ cells were enriched in *MSMP* (Microseminoprotein, Prostate Associated), *COL1A2* (Collagen Type I Alpha 2 Chain) and *COL3A1* (Collagen Type III Alpha 1 Chain), and *PLAU* (urokinase) (Fig. 4b and Supplementary File 1). Then, we studied *MDK* expression in different cell states, as well as assessed the proportion of malignant cells in each phase of the cell cycle in *MDK*^negative^ and *MDK*^positive^ cells. A higher proportion of *MDK*^positive^ cells were in the S phase, and similarly, mean MDK expression was higher in cells in the S phase (Supplementary Fig. 8). Holistically, *MDK*^positive^ cells were enriched in coagulation and epithelial-mesenchymal transition pathways (Fig. 4c).

### Gene-expression networks of *MDK*^high^ and *MDK*^low^ glioblastomas

**Fig. 5:**
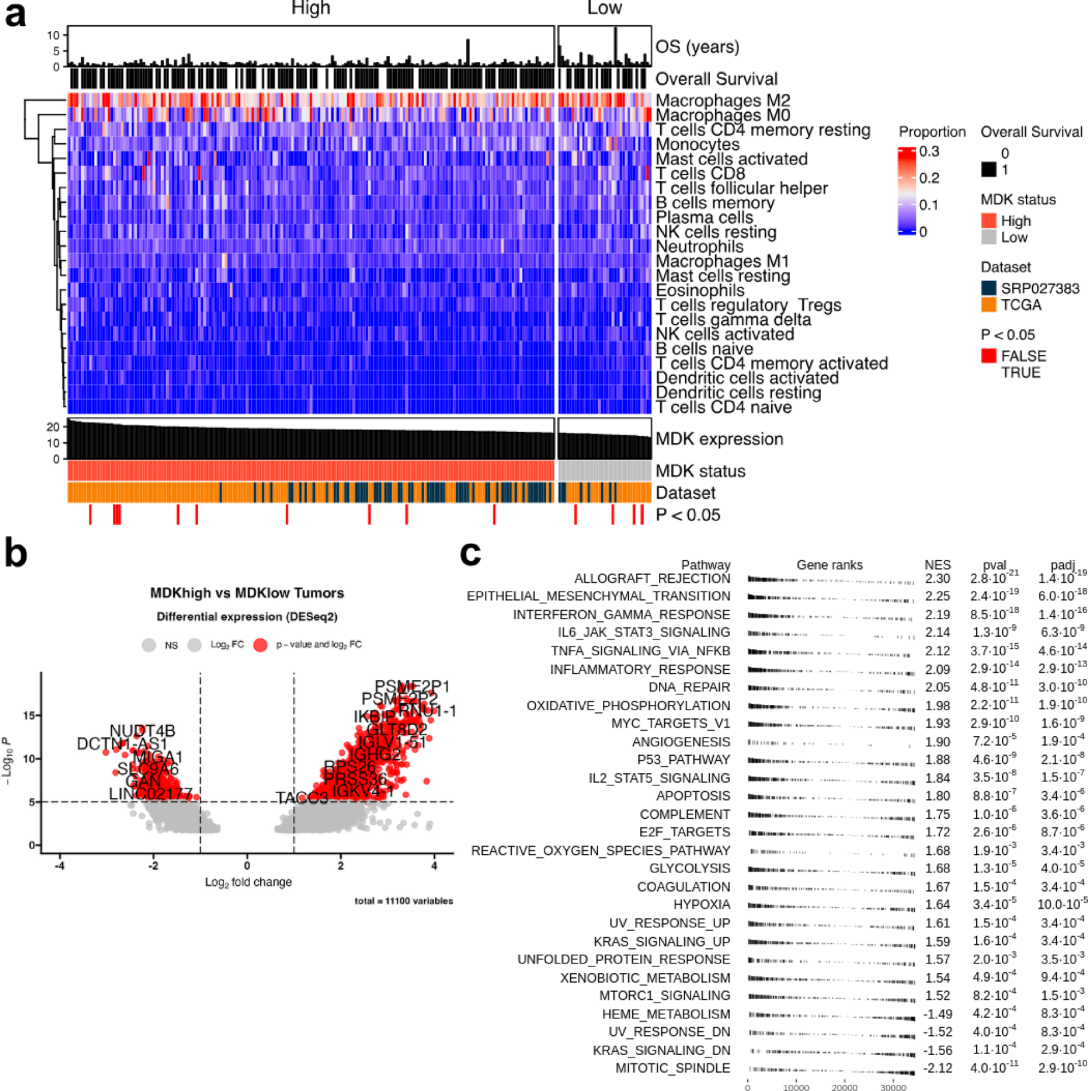
Comparison of *MDK*^high^ and *MDK*^low^ tumors. (**a**) Distribution of immune cells across the samples in *MDK*^high^ (*N* = 221) and *MDK*^low^ (*N* = 35) tumors in the combined cohort. Additionally, graphs displaying MDK expression, MDK group assignment, original dataset, and CIBERSORTx p-value are displayed below the heatmaps. Above the heatmap, overall survival is displayed as both length in years and the occurrence of death. (**b**) Differential gene expression analysis between *MDK*^high^ (*N* = 221) and *MDK*^low^ (*N* = 35) GBM tumors from the combined dataset (*N* = 256). (**c**) A table plot of gene-set enrichment analysis (Hallmark gene set) of differentially expressed genes between *MDK*^high^ (*N* = 221) and *MDK*^low^ (*N* = 35) tumors. Significantly enriched pathways are shown, with normalized enrichment scores and adjusted p-values.

To further investigate MDK-related footprint we divided the GBM from the combined dataset based on *MDK* expression into “High” (*n* = 221) and “Low” (*n* = 35) groups. The cutoff point was determined using the maxstat algorithm, as described in detail in materials and methods. We then estimated the cellular composition of GBM tumors with CIBERSORTx using the LM22 signature matrix. Surprisingly, the immune cell composition in *MDK*^high^ and *MDK*^low^ tumors remained similar, with only differences in the estimated abundance of activated NK cells (1.9% vs. 1.2%, *p* = 0.006, *MDK*^low^ and *MDK*^high^ respectively), regulatory T cells (Tregs, 1.1% vs. 1.8%, *p* = 0.016, *MDK*^low^ and *MDK*^high^ respectively) and naïve CD4 T cells (0.32% vs. 0.18%, *p* = 0.02, *MDK*^low^ and *MDK*^high^ respectively) (Fig. 5a). The estimated proportions of infiltrating cell types, together with metadata including MDK status, MDK expression, overall survival (OS) status, OS time, and source dataset, are presented as a heatmap in Fig. 5a.

We then performed differential gene expression analysis, followed by exploring the gene-expression pathways associated with *MDK*^high^ and *MDK*^low^ GBM tumors. One of the most upregulated group of genes in *MDK*^high^ tumors were C-C chemokines CCL18, CCL2, CCL13, CCL20, CCL14, CCL8, CCL26, CCL17, CCL25, CCL5, CCL3L1, CCL3, and CCL22, as well as C-X-C chemokines such as CXCL3, CXCL8, CXCL5, CXCL11, CXCL10, CXCL2, CXCL6, CXCL1, CXCL13, CXCL16, CXCL9 (Supplementary File 2). Other cytokines and protumorigenic factors such as interleukin 1a (IL-1α), interleukin 15 (IL-15), PD-L1, granzyme B, interleukin 10 (IL-10), interferon γ (IFN-γ), and granulocyte macrophage colony-stimulating factor (GM-CSF) were also upregulated in *MDK*^high^ tumors (Supplementary File 2). Pathway enrichment analysis revealed that *MDK*^high^ tumors are characterized by a mixed inflammatory profile with evidence of epithelial-mesenchymal transition, DNA repair, oxidative phosphorylation, angiogenesis, and hypoxia. (Fig. 5b, c).

### Chemokine and cytokine-inducing role of MDK

To validate the cytokine- and chemokine-rich profile observed in *MDK*^high^ GBM tumors, we performed Luminex-based secretome profiling of human macrophages, the most abundant stromal cells in GBM, and key cytokine producers. Macrophages derived from three independent donors were stimulated with physiological concentrations of recombinant MDK, GBM-conditioned media, or GBM-conditioned media supplemented with a neutralizing anti-MDK antibody.

We analyzed the secretion of 21 selected soluble factors—primarily chemokines and immunomodulatory cytokines—chosen based on their transcriptomic enrichment in *MDK*^high^ tumors. These analytes included: CCL2, MIP-1α (CCL3), CCL8, CCL13, CCL14, CCL17, CCL18, CCL26, CXCL5, CXCL6, CXCL10, CXCL11, interleukin 1 alpha (IL-1α), interleukin 10 (IL-10), interleukin 15 (IL-15), interleukin 33 (IL-33), interferon γ (IFN-γ), granulocyte-macrophage colony-stimulating factor (GM-CSF), granzyme B, programmed death-ligand 1 (PD-L1), and platelet-derived growth factor AA/BB (PDGF-AA/BB).

**Fig. 6:**
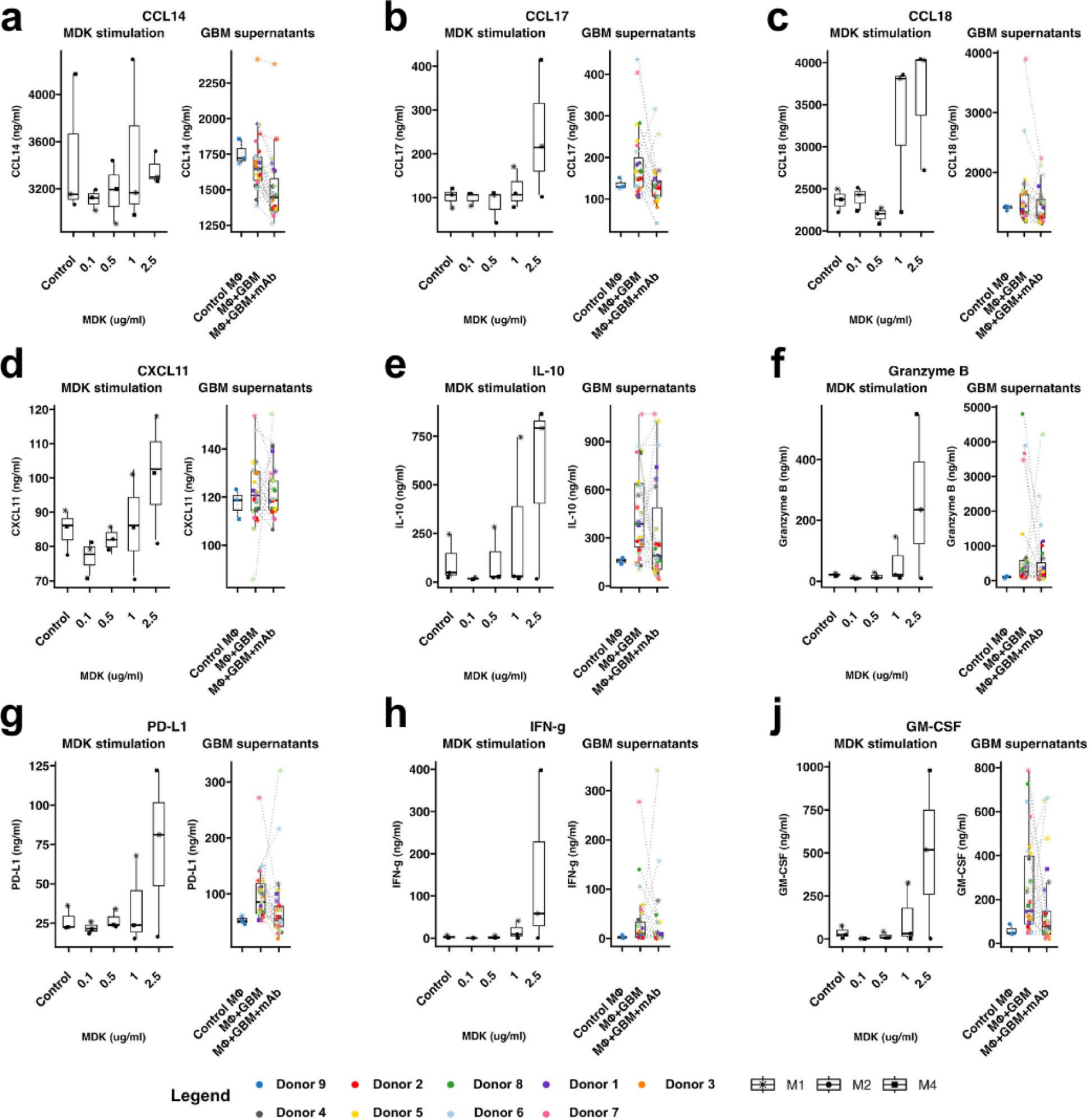
The effect of Midkine on macrophage secretome. The macrophages from different donors (*N* = 3) were stimulated with a control medium and rising physiological concentrations of midkine (0.1, 0.5, 1, and 2.5 ug/ml, the left plot of each panel) or conditioned medium from different primary GBM cell cultures (*N* = 9) with a neutralizing anti-MDK antibody added (right plot of each panel), with GBM culture medium serving as a control. The secretion of the following analytes was analyzed: (**a**) CCL14, (**b**) CCL17, (**c**) CCL18, (**d**) CXCL11, (**e**) IL-10, (**f**) Granzyme B, (**g**) PD-L1, (**h**) IFN-y, and (**i**) GM-CSF. The results are presented as bar plots with a median and a dot plot with each data point. The concentration of analytes is displayed in ng/ml.

Stimulation with recombinant MDK led to a marked increase in the secretion of chemokines such as MIP-1α (CCL3), CCL17, CCL18, CXCL5, CXCL10, and CXCL11, as well as immunomodulatory and proinflammatory factors including IL-1α, IL-15, IL-33, IL-10, GM-CSF, granzyme B, PD-L1, PDGF-AA/BB, and IFN-γ (Fig. 6, Supplementary Figs. 9 & 10). These effects were dose-dependent and proportional to the concentration of MDK.

Exposure to GBM-conditioned media also significantly increased the secretion of several factors, including CCL17, CXCL11, IL-10, granzyme B, PD-L1, IFN-γ, GM-CSF, CCL13, CXCL5, CXCL6, CXCL10, PDGF-AA/BB, IL-1α, IL-15, and IL-33 (Fig. 6, Supplementary Figs. 9 & 10). Notably, this upregulation was partially reversed by the addition of a neutralizing anti-MDK antibody, implicating MDK as an effector in mediating these responses. Interestingly, GBM-conditioned media alone did not elevate secretion of CCL14, CCL18, or MIP-1α; however, their levels decreased upon anti-MDK treatment, suggesting that while mixed factors affect their expression, MDK also plays a role in their upregulation. In contrast, MDK stimulation had no apparent effect on the secretion of CCL2, CCL8, CCL13, or CCL26 within the concentration range tested (Fig. 6, Supplementary Figs. 9 & 10).

Taken together, both the recombinant MDK stimulation and the GBM-conditioned media experiments point to MDK as a potent modulator of macrophage function, promoting the secretion of multiple immunologically relevant soluble factors—most notably CCL17, CXCL10, PD-L1, IFN-γ, GM-CSF, CXCL5, and to a lesser extent granzyme B, CCL18, CXCL6, CXCL11 (Fig. 6, Supplementary Figs. 9 & 10). Importantly, all of the abovementioned factors were significantly upregulated in the differential gene expression analysis in *MDK*^high^ GBM tumors.

### Mutational and methylation profiles of *MDK*^high^ and *MDK*^low^ tumors

**Fig. 7:**
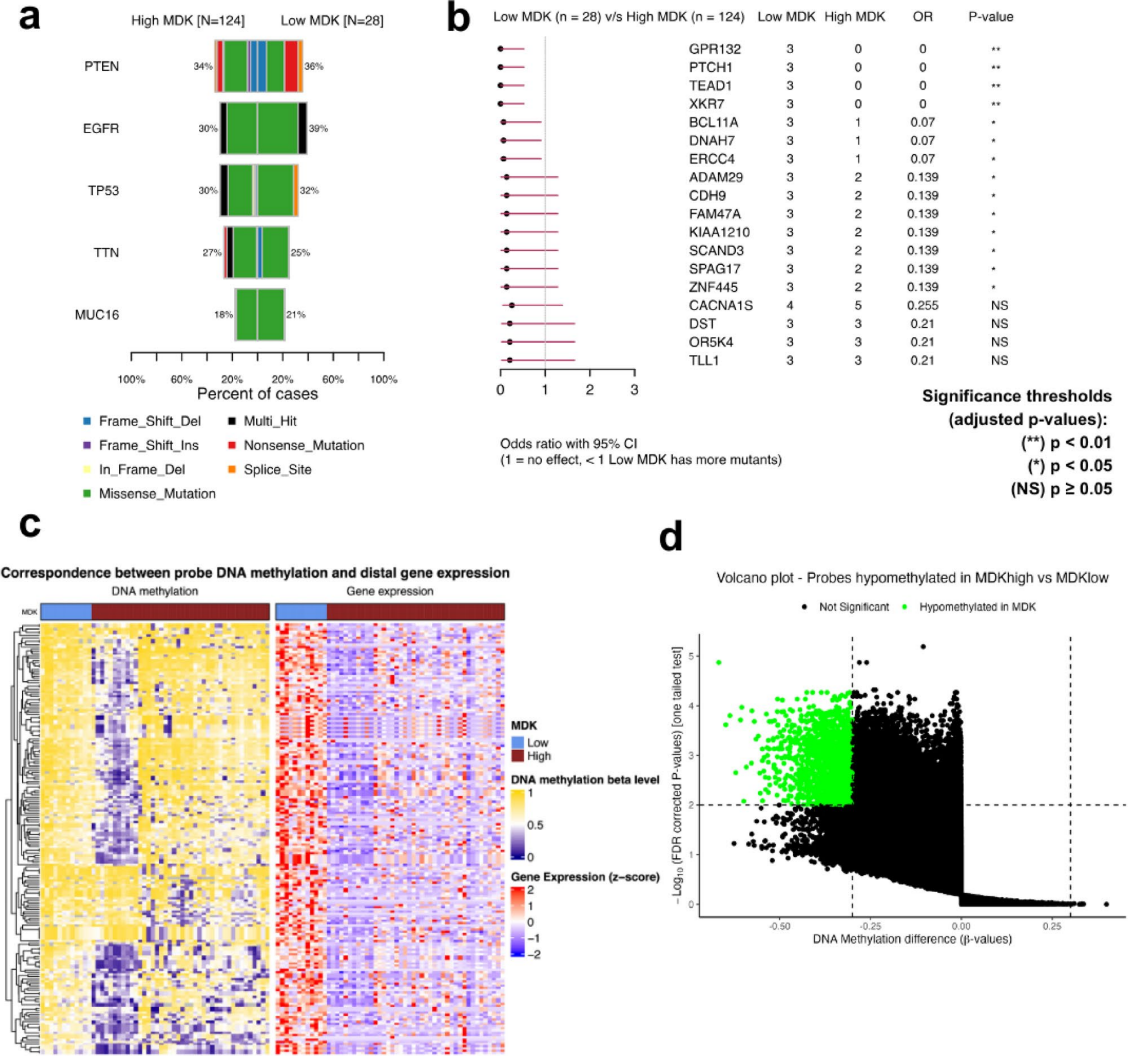
Somatic mutations in *MDK*^high^ and *MDK*^low^ tumors. (**a**) The most frequently mutated genes in *MDK*^high^ (*N* = 124) and *MDK*^low^ (*N* = 28) tumors. (**b**) Differentially mutated genes between *MDK*^high^ (*N* = 124) and *MDK*^low^ (*N* = 28) displayed as a forest plot. Bars indicate a 95% confidence interval of the odds ratio. The adjacent table includes the number of samples in both groups with the mutations in the highlighted gene. Statistical significance was assessed by Fisher’s exact test. (**c**) Differentially methylated probes in *MDK*^high^ (*N* = 42) and *MDK*^low^ (*N* = 12) tumors. (d) Heatmap of DNA methylation and linked mRNA expression patterns in *MDK*^high^ (*N* = 42) and *MDK*^low^ (*N* = 12) tumors in the TCGA-GBM cohort.

We next examined the somatic mutational profiles of *MDK*^high^ and *MDK*^low^ tumors. *PTEN*, *EGFR*, and *TP53* were, predictably, the most frequently point-mutated genes. (Fig. 7a). While their prevalence remained relatively similar in both *MDK*^high^ and *MDK*^low^ tumors, *MDK*^low^ tumors were enriched in GPR132, PTCH1, TEAD1, and XKR7 mutations, which were exclusively identified in that group at a frequency of 11% (vs. 0%) (Fig. 7b).

Finally, we investigated the genome-wide DNA methylation patterns between *MDK*^high^ and *MDK*^low^ tumors. The latter were characterized by a unique hypermethylated genome, as indicated by the vast majority of probes with only sporadic islands of mild hypomethylation (Fig. 7c). Conversely, *MDK*^high^ tumors were a heterogeneous group with a large set of hypomethylated probes, where the methylation pattern divided the samples into two groups based on four different sets of probes (Fig. 7c & d).

## Discussion

In this study, we harnessed the power of a large, harmonized RNA-seq dataset complemented with primary tumor samples and matched primary cell cultures to comprehensively characterize midkine (MDK) expression, secretion, and MDK-associated multiomic footprint in grade IV *IDH*^wildtype^ gliomas (glioblastomas, GBM). We showed that gliomas express and secrete MDK in excessive quantities with higher expression in *IDH*^wildtype^ gliomas. Our study also indicates that MDK expression in *IDH*^wildtype^ gliomas correlates with tumor grade, potentially reflecting selective pressure favoring the ENST00000395566 isoform, and that high midkine expression is associated with a poor prognosis in *IDH*^wildtype^ grade IV gliomas (GBM). Utilization of a dataset combined from ethnically and geographically diverse cohorts ensures the robustness and clinical applicability of the expression patterns and survival analyses. Its apparent prognostic effect and heterogenous expression in GBM allowed for the identification of a large group of *MDK*^high^ tumors constituting 75% of all GBM, representing a cohort potentially benefiting from MDK-targeting treatment. Additionally, identification of the ENST00000395566 MDK isoform as the most prevalent and selectively increased along the glioma grade shows that while the truncated form of MDK has been proposed to be specific to certain tumors, in gliomas, the full-length transcript is the most prevalent and favored^59,60^. The ENST00000395566 transcript corresponds to the MANE-selected canonical isoform and contains all functional domains of midkine, including heparin-binding and growth factor activity regions. In contrast, some less abundant isoforms may lack domains necessary for full signaling capacity, suggesting that selection pressure in GBM favors the fully functional transcript.

The mechanism underlying the widespread yet variable MDK expression in GBM has not been elucidated. Originally, *MDK* has been described as a retinoic acid-inducible gene^34^. Given that retinoic acid signaling is commonly overactivated in high-grade gliomas and correlates with adverse prognosis, our findings suggest a plausible involvement of MDK in mediating these effects, warranting further mechanistic investigation. We have found that retinol binding protein 1 (*RBP1)* expression, in fact, strongly correlates with *MDK* expression in our combined dataset. Another possible mechanism is related to estrogen, as *MDK* is an estrogen-responsive gene^61^. We show that *MDK* expression weakly correlates with the HALLMARK_ESTROGEN_RESPONSE_LATE gene signature. Hypoxia can further contribute to increased MDK expression due to hypoxia-responsive element in *MDK* promoter^33,62^. In our study, *MDK* expression also weakly correlates with hypoxia signature, suggesting a possible contribution of retinoic acid signaling, potentially acting in concert with other pathways.

Analysis on a single-cell level revealed that *MDK* expression is associated with increased cell proliferation as well as pathways related to epithelial-mesenchymal transition (EMT) and coagulation. The EMT increases the mobility potential of malignant cells and is a critical step in cancer invasion and metastasis. The increased proliferation, migration, and EMT are likely mediated through MDK-induced (anaplastic lymphoma kinase) ALK and MET pathway activation^63–65^. High expression of pro-coagulation factors and matrix metalloproteinases can contribute to dysfunctional microvasculature, one of the hallmarks of glioblastoma^66,67^. On a tissue level, we found that *MDK*^high^ tumors are enriched in pathways related to epithelial-mesenchymal transition, DNA repair, oxidative phosphorylation, angiogenesis, and hypoxia. Additionally, *MDK*^low^ tumors were enriched in GPR132, PTCH1, TEAD1, and XKR7 mutations, which were not identified in *MDK*^high^ tumors. Mutations in, e.g., PTCH1 are associated with favorable response to checkpoint inhibitor therapy, which might affect the treatment of *MDK*^low^ tumors in the future^68^.

MDK is an established regulator of cell migration. It was shown to directly facilitate the migration of monocytes and polymorphonuclear neutrophils (PMNs) in soluble and substrate-bound forms. Additionally, MDK was shown to induce chemokine secretion in human T cells^32,69,70^. In our study, we found that most highly expressed group of genes in *MDK*^high^ tumors in our study were chemokines including C-C chemokines such as CCL18, CCL2, CCL13, CCL20, CCL14, CCL8, CCL26, CCL17, CCL25, CCL5, CCL3L1, CCL3, and CCL22, as well as C-X-C chemokines such as CXCL1-3, CXCL5-6, CXCL8-11, CXCL13, CXCL16, which are known to attract TAMs, protumorigenic neutrophils and immature NK cells to the TME, as well as synergistically promoting angiogenesis along with MDK^71–73^. Interestingly, chemokines were not upregulated in *MDK*^positive^ GBM cells, indicating that the chemokine-inducing effect of MDK is mediated by tumor-infiltrating cells, as suggested by a recent study by Guo et al.^32^. *MDK*^high^ tumors were also characterized by upregulated expression of interleukin 1a (IL-1α), interleukin 15 (IL-15), PD-L1, granzyme B, interleukin 10 (IL-10), interferon-γ (IFN-γ), and granulocyte macrophage colony-stimulating factor (GM-CSF).

Macrophages are key cytokine producers that shape their surrounding microenvironment. In glioblastoma (GBM), they constitute the predominant stromal component, contributing to multiple tumor-promoting processes^74^. Here, we provide functional evidence that GBM-secreted MDK modulates the macrophage secretome, skewing it toward a pro-tumorigenic and immunosuppressive phenotype. Using Luminex profiling of macrophages from three independent donors, we obtained data supporting the results of differential gene expression analysis between *MDK*^high^ and *MDK*^low^ GBM tumors, and identified 20 soluble factors modulated by MDK, including chemokines: MIP-1a (CCL3), CCL13, CCL14, CCL17, CCL18, CXCL5, CXCL6, CXCL10, CXCL10, CXCL11, cytokines interleukin 1 alpha (IL-1α), interleukin 10 (IL-10), interleukin 15 (IL-15), interleukin 33 (IL-33), interferon γ (IFN-γ), granulocyte-macrophage colony-stimulating factor (GM-CSF), platelet-derived growth factor AA/BB (PDGF-AA/BB), and other soluble factors such as programmed death-ligand 1 (PD-L1), granzyme B.

Among the prominently MDK-induced chemokines, CCL17, a known CCR4 ligand, is associated with the recruitment of regulatory T cells (Tregs) and immunosuppression in various tumors. Our RNA-seq deconvolution analysis revealed a higher proportion of Tregs in *MDK*^high^ tumors compared to *MDK*^low^, implicating the role of MDK-induced CCL17 in promoting a Treg-enriched microenvironment^75^. CCL18, predominantly expressed by tumor-associated macrophages, has been shown to promote epithelial-to-mesenchymal transition (EMT), tumor cell migration, and invasion^76^. Using a humanized glioma model, CCL18 was recently demonstrated to drive glioma progression via CCR8-ACP5 signaling^77^. IL-10, an anti-inflammatory cytokine produced by myeloid cells, has been implicated in mediating T-cell dysfunction in the GBM microenvironment^78^. GM-CSF, another MDK-induced cytokine, supports the expansion of immunosuppressive myeloid populations in GBM through IL-4R alpha and is associated with the tumor’s characteristic lymphopenia and systemic immune dysfunction^79,80^. Furthermore, PD-L1, a key immune checkpoint molecule, was significantly upregulated in response to MDK, potentially contributing to T-cell exhaustion in GBM^81^. Finally, Granzyme B, traditionally associated with cytotoxic lymphocytes, was also increased in MDK-stimulated macrophages. Emerging evidence suggests it may participate in tissue remodeling and immune modulation within the tumor microenvironment^82^. Crucially, the use of a neutralizing anti-MDK antibody indicated that these effects were MDK-specific: the upregulation of these factors in response to GBM-conditioned media was partially reversed upon MDK blockade. Taken together, our findings identify MDK as a potent regulator of the macrophage secretome, which may indicate that it also has a similar effect in tumor-associated macrophages (TAM) in GBM by promoting the expression of factors that collectively enhance immune suppression, tumor cell invasion, and therapy resistance.

At the single-cell level, the most upregulated genes in *MDK*^positive^ cells were *MSMP* (microseminoprotein), *PLAU* (urokinase), and the collagen-encoding genes *COL1A2* and *COL3A1*. *MSMP* has been implicated in the chemotaxis of CCR2-expressing cells and resistance to anti-VEGF therapy in ovarian cancer^83,84^. While its role in GBM remains unclear, it has been detected at both mRNA and protein levels and is suggested to be associated with tumor stemness^85^. *PLAU* and its receptor *PLAUR* have been shown to induce a mesenchymal gene expression signature in GBM cells, promoting tumor cell survival and correlating with poor prognosis^86^. Collagens and extracellular matrix components are critical in GBM biology; inhibition of *COL1A2* and *COL3A1* significantly suppresses cell proliferation, colony formation, and invasiveness. Notably, *COL1A2* expression is reduced by phosphoinositide 3-kinase (PI3K) inhibition, and since MDK signals through the PI3K pathway, this may explain the observed upregulation of *COL1A2* in MDK-positive cells. Earlier studies also support the role of MDK in promoting collagen expression^87–89^. Conversely, *HBB* (hemoglobin subunit beta) and *LOX* (lysyl oxidase) were among the most downregulated genes in *MDK*^positive^ cells. *HBB*, encoding beta-globin, has been reported to act as both a tumor suppressor and promoter, depending on the cancer type^90,91^. The downregulation of *LOX* is unexpected, given its established role in enhancing glioma invasiveness via extracellular matrix crosslinking and remodeling through enhancing epidermal growth factor receptor (EGFR) signaling^92–94^. We hypothesize that in *MDK*^positive^ cells, where the MDK receptor LRP-1 (low density lipoprotein receptor-related protein 1) stabilizes activated EGFR, the EGFR-entrapping function of LOX may become redundant, potentially explaining its decreased expression^95^. Further investigation is warranted to elucidate the interplay between MDK and LOX signaling in GBM.

All of the above-mentioned rationales may explain the lower survival rate and worse prognostic outcomes in the *MDK*^high^ group, indicating that MDK plays a central role in GBM biology, and its inhibition might be a feasible strategy to increase the treatment efficacy of larger treatment regimens through inhibition of GBM cell proliferation and EMT as well as rebalancing of the tumor microenvironment. After MDK was discovered in 1988, it soon became a spotlight target in cancer research^34,96^. However, the discovered complexity of MDK signaling and its receptor network halted further research due to a need for advanced molecular biology techniques that were yet unavailable^65^. Recent advancements in molecular biology and genetic engineering techniques have made targeting MDK feasible. Several *MDK* inhibition strategies, including MDK inhibitor iMDK, siRNA, and CRISPR-Cas9 liposomes, were evaluated in preclinical studies^16,31,97^. These initial reports show that MDK inhibition restores temozolomide (TMZ) sensitivity and attenuates the growth of GBM cell lines and patient-derived GBM models^16,31,97^.

A particularly promising approach is based on crizotinib, an orally available 1st generation ATP-competitive ALK and c-MET dual inhibitor^98,99^. After encouraging preclinical reports showing potent antitumor activity, a phase I clinical study (NCT02270034) has been launched and completed^15,100–102^. The treatment was well-tolerated and the initial efficacy report showed that crizotinib combined with standard of care – TMZ (temzolomide) + radiotherapy (RT), resulted in a median progression-free survival (PFS) of 10.7 months and a median overall survival (OS) of 22.6 months^102^. While no direct comparison with only TMZ + RT was made, both OS and PFS were higher than expected and higher than in other studies combining other small-molecule inhibitors with TMZ and RT^3,103–107^. Notably, the pharmacotherapy was initiated 5.3 weeks (median) after surgery, and post-treatment MDK serum levels were similar to those observed at baseline. Since MDK is an injury-inducible cytokine, its levels most likely rise after surgery-induced tissue damage, promoting survival and proliferation of residual GBM cells^20,96^. This indicates that any MDK-targeted therapy should be initiated at the diagnosis if it does not interfere with surgery and post-surgical care. A possible prognostic value of serum MDK concentrations has not been observed. We show that, while *MDK* mRNA levels have robust prognostic value, they do not correlate to MDK serum levels. However, both our study and the NCT02270034 clinical trial are limited by a small sample size, and further research is needed to evaluate the correlation of MDK mRNA expression and MDK serum levels and a possible prognostic value of the latter.

This study has several limitations. While our aim was to conduct an explorative analysis of MDK expression patterns, survival associations, and its putative effects on GBM biology, much of our work is based on in silico analyses. As such, inferred pathway activation, cell-type deconvolution, and expression correlations are hypothesis-generating and require further validation both in vivo through manipulating *MDK*, as well as in additional ex vivo systems. Additionally, macrophage-stimulation experiments were limited to three macrophage donors, which may not fully capture inter-individual variability in immune responses. While our transcriptomic analyses were based on large, geographically, and ethnically diverse datasets corrected for batch effects, functional validation (including serum and conditioned media experiments) was conducted on a relatively narrow patient cohort, limiting generalizability. Importantly, our study does not include in vivo validation of MDK-targeted therapies in GBM models, a critical step before clinical translation. Despite these limitations, we addressed potential biases by integrating multiple independent datasets, applying rigorous statistical controls, and complementing in silico findings with functional macrophage assays and protein-level analyses.

In summary, our study’s combination of computational and functional analyses provides novel insight into MDK as a key contributor to a distinct, aggressive glioblastoma phenotype. We characterized MDK expression patterns in a wide range of gliomas from ethnically different cohorts and revealed coordinated changes in its expression and isoform proportions. We showed that the primary source of MDK in GBM TME are the malignant cells, producing MDK likely upon retinoic acid-, estrogen-, and hypoxia-induced signaling. Multiomic analysis shows that MDK has strong, pleiotropic protumorigenic effects excreted through both direct effects on malignant cells and indirect through reshaping the TME, as confirmed by uncovering MDK’s potential to induce protumorigenic cytokine and chemokine profile in macrophages. These facts underline the potential for MDK inhibition in a large subset of GBM patients, particularly in combination treatment.

## Supplementary Information

Below is the link to the electronic supplementary material.

Supplementary Material 1

Supplementary Material 2

Supplementary Material 3

## Data Availability

Sequence data have been deposited at the European Genome-phenome Archive (EGA), hosted by the EBI and the CRG, under accession number EGAS00001006267. M.L. will provide the code used for data analysis upon request from the corresponding author.

## References

[1] Hanif, F., Muzaffar, K., Perveen, K., Malhi, S. M. & Simjee, S. U. Glioblastoma multiforme: A review of its epidemiology and pathogenesis through clinical presentation and treatment. *Asian Pac. J. Cancer Prev.* **18**, 3–9 (2017). 28239999 10.22034/APJCP.2017.18.1.3 PMC5563115

[2] Davis, M. E. & Glioblastoma Overview of disease and treatment. *Clin. J. Oncol. Nurs.* **20**, S2–S8 (2016). 27668386 10.1188/16.CJON.S1.2-8 PMC5123811

[3] Louis, D. N. et al. The 2021 WHO classification of tumors of the central nervous system: a summary. *Neuro Oncol.* **23**, 1231–1251 (2021). 34185076 10.1093/neuonc/noab106 PMC8328013

[4] Poon, M. T. C., Sudlow, C. L. M., Figueroa, J. D. & Brennan, P. M. Longer-term (≥ 2 years) survival in patients with glioblastoma in population-based studies pre- and post-2005: a systematic review and meta-analysis. *Sci. Rep.* **10**, 11622 (2020). 32669604 10.1038/s41598-020-68011-4 PMC7363854

[5] Di Nunno, V., Gatto, L., Tosoni, A., Bartolini, S. & Franceschi, E. Implications of BRAF V600E mutation in gliomas: molecular considerations, prognostic value and treatment evolution. *Front. Oncol.* **12**, 1067252 (2023). 36686797 10.3389/fonc.2022.1067252 PMC9846085

[6] Kanemaru, Y. et al. Dramatic response of BRAF V600E-mutant epithelioid glioblastoma to combination therapy with BRAF and MEK inhibitor: establishment and xenograft of a cell line to predict clinical efficacy. *Acta Neuropathol. Commun.* **7**, 119 (2019). 31345255 10.1186/s40478-019-0774-7 PMC6659204

[7] Pearson, J. R. D. et al. Immune escape in glioblastoma multiforme and the adaptation of immunotherapies for treatment. *Frontiers Immunology* **11**, 582106 (2020). 10.3389/fimmu.2020.582106 PMC7594513 33178210

[8] DeCordova, S. et al. Molecular heterogeneity and immunosuppressive microenvironment in glioblastoma. *Frontiers Immunology* **11**, 1402 (2020). 10.3389/fimmu.2020.01402 PMC7379131 32765498

[9] McLendon, R. et al. Comprehensive genomic characterization defines human glioblastoma genes and core pathways. *Nature* **455**, 1061–1068 (2008). 18772890 10.1038/nature07385 PMC2671642

[10] Brennan, C. W. et al. The somatic genomic landscape of glioblastoma. *Cell* **155**, 462–477 (2013). 24120142 10.1016/j.cell.2013.09.034 PMC3910500

[11] Inda, M. M. et al. Tumor heterogeneity is an active process maintained by a mutant EGFR-induced cytokine circuit in glioblastoma. *Genes Dev.* **24**, 1731–1745 (2010). 20713517 10.1101/gad.1890510 PMC2922502

[12] Domagala, J. et al. The tumor Microenvironment—A metabolic obstacle to NK cells’ activity. *Cancers* **12**, 3542 (2020). 33260925 10.3390/cancers12123542 PMC7761432

[13] Neumaier, E. E., Rothhammer, V. & Linnerbauer, M. The role of midkine in health and disease. *Frontiers Immunology* **14**, 1310094 (2023). 10.3389/fimmu.2023.1310094 PMC10720637 38098484

[14] Lorente, M. et al. Stimulation of the midkine/alk axis renders glioma cells resistant to cannabinoid antitumoral action. *Cell. Death Differ.* **18**, 959–973 (2011). 21233844 10.1038/cdd.2010.170 PMC3131933

[15] López-Valero, I. et al. Midkine signaling maintains the self-renewal and tumorigenic capacity of glioma initiating cells. *Theranostics* **10**, 5120–5136 (2020). 32308772 10.7150/thno.41450 PMC7163450

[16] Hu, B. et al. Midkine promotes glioblastoma progression via PI3K-Akt signaling. *Cancer Cell. Int.* **21**, 509 (2021). 34556138 10.1186/s12935-021-02212-3 PMC8461913

[17] Satoh, J. et al. Midkine that promotes survival of fetal human neurons is produced by fetal human astrocytes in culture. *Brain Res. Dev. Brain Res.* **75**, 201–205 (1993). 8261612 10.1016/0165-3806(93)90024-5

[18] Kadomatsu, K., Huang, R. P., Suganuma, T., Murata, F. & Muramatsu, T. A retinoic acid responsive gene MK found in the teratocarcinoma system is expressed in spatially and temporally controlled manner during mouse embryogenesis. *J. Cell. Biol.* **110**, 607–616 (1990). 1689730 10.1083/jcb.110.3.607 PMC2116029

[19] Muramatsu, T. Midkine, a heparin-binding cytokine with multiple roles in development, repair and diseases. *Proc. Jpn Acad. Ser. B Phys. Biol. Sci.* **86**, 410–425 (2010). 20431264 10.2183/pjab.86.410 PMC3417803

[20] Ross-Munro, E. et al. Midkine: the who, what, where, and when of a promising neurotrophic therapy for perinatal brain injury. *Front. Neurol.* **11**, 568814 (2020). 33193008 10.3389/fneur.2020.568814 PMC7642484

[21] Muramatsu, H. et al. alpha4beta1- and alpha6beta1-integrins are functional receptors for midkine, a heparin-binding growth factor. *J. Cell. Sci.* **117**, 5405–5415 (2004). 15466886 10.1242/jcs.01423

[22] Stoica, G. E. et al. Midkine binds to anaplastic lymphoma kinase (ALK) and acts as a growth factor for different cell Types *. *J. Biol. Chem.* **277**, 35990–35998 (2002). 12122009 10.1074/jbc.M205749200

[23] Mitsiadis, T. A. et al. Expression of the heparin-binding cytokines, midkine (MK) and HB-GAM (pleiotrophin) is associated with epithelial-mesenchymal interactions during fetal development and organogenesis. *Development* **121**, 37–51 (1995). 7867507 10.1242/dev.121.1.37

[24] Huang, Y. et al. Midkine induces epithelial-mesenchymal transition through Notch2/Jak2-Stat3 signaling in human keratinocytes. *Cell. Cycle*. **7**, 1613–1622 (2008). 18469519 10.4161/cc.7.11.5952

[25] Muramatsu, H. et al. LDL receptor-related protein as a component of the midkine receptor. *Biochem. Biophys. Res. Commun.* **270**, 936–941 (2000). 10772929 10.1006/bbrc.2000.2549

[26] Maeda, N. et al. A receptor-like protein-tyrosine phosphatase ptpzeta/rptpbeta binds a heparin-binding growth factor midkine. Involvement of arginine 78 of midkine in the high affinity binding to PTPzeta. *J. Biol. Chem.* **274**, 12474–12479 (1999). 10212223 10.1074/jbc.274.18.12474

[27] Filippou, P. S., Karagiannis, G. S. & Constantinidou, A. Midkine (MDK) growth factor: a key player in cancer progression and a promising therapeutic target. *Oncogene* **39**, 2040–2054 (2020). 31801970 10.1038/s41388-019-1124-8

[28] Hanahan, D. Hallmarks of cancer: new dimensions. *Cancer Discov.* **12**, 31–46 (2022). 35022204 10.1158/2159-8290.CD-21-1059

[29] Mishima, K. et al. Increased expression of midkine during the progression of human Astrocytomas. *Neurosci. Lett.* **233**, 29–32 (1997). 9324232 10.1016/s0304-3940(97)00619-8

[30] Cheng, Y. P. et al. Midkine expression in high grade gliomas: correlation of this novel marker with proliferation and survival in human gliomas. *Surg. Neurol. Int.* **5**, 78 (2014). 24949221 10.4103/2152-7806.133205 PMC4061577

[31] Yu, X. et al. MDK induces Temozolomide resistance in glioblastoma by promoting cancer stem-like properties. *Am. J. Cancer Res.* **12**, 4825–4839 (2022). 36381313 PMC9641408

[32] Guo, X. et al. Midkine activation of CD8 + T cells establishes a neuron–immune–cancer axis responsible for low-grade glioma growth. *Nat. Commun.* **11**, 2177 (2020). 32358581 10.1038/s41467-020-15770-3 PMC7195398

[33] Xia, M., Tong, S. & Gao, L. Identification of MDK as a Hypoxia- and Epithelial–Mesenchymal Transition-Related gene biomarker of glioblastoma based on a novel risk model and in vitro experiments. *Biomedicines* **12**, 92 (2024). 38255198 10.3390/biomedicines12010092 PMC10813330

[34] Kadomatsu, K., Tomomura, M. & Muramatsu, T. cDNA cloning and sequencing of a new gene intensely expressed in early differentiation stages of embryonal carcinoma cells and in mid-gestation period of mouse embryogenesis. *Biochem. Biophys. Res. Commun.* **151**, 1312–1318 (1988). 3355557 10.1016/s0006-291x(88)80505-9

[35] Tang, S. L., Gao, Y. L. & Chen, X. B. Wnt/β-catenin up-regulates midkine expression in glioma cells. *Int. J. Clin. Exp. Med.* **8**, 12644–12649 (2015). 26550177 PMC4612862

[36] Gozdz, A. et al. Preservation of the hypoxic transcriptome in glioblastoma Patient-Derived cell lines maintained at Lowered oxygen tension. *Cancers (Basel)*. **14**, 4852 (2022). 36230775 10.3390/cancers14194852 PMC9564145

[37] Leelatian, N. et al. Preparing viable single cells from human tissue and tumors for cytomic analysis. *Curr. Protoc. Mol. Biol.* **118**, 25C11–25C123 (2017). 10.1002/cpmb.37 PMC5518778 28369679

[38] Kovaka, S. et al. Transcriptome assembly from long-read RNA-seq alignments with StringTie2. *Genome Biol.* **20**, 278 (2019). 31842956 10.1186/s13059-019-1910-1 PMC6912988

[39] Kim, D., Paggi, J. M., Park, C., Bennett, C. & Salzberg, S. L. Graph-based genome alignment and genotyping with HISAT2 and HISAT-genotype. *Nat. Biotechnol.* **37**, 907–915 (2019). 31375807 10.1038/s41587-019-0201-4 PMC7605509

[40] Wickham, H. et al. Welcome to the tidyverse. *J. Open. Source Softw.* **4**, 1686 (2019).

[41] Alquicira-Hernandez, J. & Powell, J. E. Nebulosa recovers single-cell gene expression signals by kernel density Estimation. *Bioinformatics* **37**, 2485–2487 (2021). 33459785 10.1093/bioinformatics/btab003

[42] Gu, Z., Eils, R. & Schlesner, M. Complex heatmaps reveal patterns and correlations in multidimensional genomic data. *Bioinformatics* **32**, 2847–2849 (2016). 10.1093/bioinformatics/btw313 27207943

[43] Collado-Torres, L. et al. Reproducible RNA-seq analysis using recount2. *Nat. Biotechnol.* **35**, 319–321 (2017). 28398307 10.1038/nbt.3838 PMC6742427

[44] Zhao, Z. et al. Chinese glioma genome atlas (CGGA): A comprehensive resource with functional genomic data from Chinese glioma patients. *Genom. Proteom. Bioinform.* **19**, 1–12 (2021). 10.1016/j.gpb.2020.10.005 PMC8498921 33662628

[45] Gill, B. J. et al. MRI-localized biopsies reveal subtype-specific differences in molecular and cellular composition at the margins of glioblastoma. *Proc. Natl. Acad. Sci. U S A*. **111**, 12550–12555 (2014). 25114226 10.1073/pnas.1405839111 PMC4151734

[46] The Cancer Genome Atlas Research Network. Comprehensive, integrative genomic analysis of diffuse Lower-Grade gliomas. *N. Engl. J. Med.* **372**, 2481–2498 (2015). 26061751 10.1056/NEJMoa1402121 PMC4530011

[47] Zhang, Y. et al. Purification and characterization of progenitor and mature human astrocytes reveals transcriptional and functional differences with mouse. *Neuron* **89**, 37–53 (2016). 26687838 10.1016/j.neuron.2015.11.013 PMC4707064

[48] Zhang, Y. et al. An RNA-Sequencing transcriptome and splicing database of glia, neurons, and vascular cells of the cerebral cortex. *J. Neurosci.* **34**, 11929–11947 (2014). 25186741 10.1523/JNEUROSCI.1860-14.2014 PMC4152602

[49] Leek, J. T., Johnson, W. E., Parker, H. S., Jaffe, A. E. & Storey, J. D. The Sva package for removing batch effects and other unwanted variation in high-throughput experiments. *Bioinformatics* **28**, 882–883 (2012). 22257669 10.1093/bioinformatics/bts034 PMC3307112

[50] Neftel, C. et al. An integrative model of cellular states, plasticity, and genetics for glioblastoma. *Cell* **178**, 835–849e21 (2019). 31327527 10.1016/j.cell.2019.06.024 PMC6703186

[51] Love, M. I., Huber, W. & Anders, S. Moderated Estimation of fold change and dispersion for RNA-seq data with DESeq2. *Genome Biol.* **15**, 550 (2014). 25516281 10.1186/s13059-014-0550-8 PMC4302049

[52] Ignatiadis, N., Klaus, B., Zaugg, J. B. & Huber, W. Data-driven hypothesis weighting increases detection power in genome-scale multiple testing. *Nat. Methods*. **13**, 577–580 (2016). 27240256 10.1038/nmeth.3885 PMC4930141

[53] Korotkevich, G. et al. Fast gene set enrichment analysis. 060012 Preprint at (2021). 10.1101/060012

[54] Newman, A. M. et al. Determining cell type abundance and expression from bulk tissues with digital cytometry. *Nat. Biotechnol.* **37**, 773–782 (2019). 31061481 10.1038/s41587-019-0114-2 PMC6610714

[55] Silva, T. C. et al. *TCGA Workflow*: analyze cancer genomics and epigenomics data using bioconductor packages. Preprint at https://doi.org/ (2016). 10.12688/f1000research.8923.2 10.12688/f1000research.8923.2 PMC5302158 28232861

[56] Silva, T. C. et al. ELMER v.2: an r/bioconductor package to reconstruct gene regulatory networks from DNA methylation and transcriptome profiles. *Bioinformatics* **35**, 1974–1977 (2019). 30364927 10.1093/bioinformatics/bty902 PMC6546131

[57] Hoffman, P. Seurat: Tools for Single Cell Genomics. (2022).

[58] Kowalczyk, M. S. et al. Single-cell RNA-seq reveals changes in cell cycle and differentiation programs upon aging of hematopoietic stem cells. *Genome Res.* **25**, 1860–1872 (2015). 26430063 10.1101/gr.192237.115 PMC4665007

[59] Aridome, K. et al. Truncated midkine as a marker of diagnosis and detection of nodal metastases in Gastrointestinal carcinomas. *Br. J. Cancer*. **78**, 472–477 (1998). 9716029 10.1038/bjc.1998.517 PMC2063076

[60] Miyashiro, I. et al. Expression of truncated midkine in human colorectal cancers. *Cancer Lett.* **106**, 287–291 (1996). 8844985 10.1016/0304-3835(96)04333-9

[61] Zhao, G. et al. ERβ-mediated estradiol enhances epithelial mesenchymal transition of lung adenocarcinoma through increasing transcription of midkine. *Mol. Endocrinol.* **26**, 1304–1315 (2012). 22669742 10.1210/me.2012-1028 PMC5416977

[62] Weckbach, L. T. et al. Midkine acts as proangiogenic cytokine in hypoxia-induced angiogenesis. *Am. J. Physiol. Heart Circ. Physiol.* **303**, H429–438 (2012). 22707563 10.1152/ajpheart.00934.2011

[63] Wellstein, A. ALK receptor activation, ligands and therapeutic targeting in glioblastoma and in other cancers. *Front Oncol*. **2**, 192 (2012). 10.3389/fonc.2012.00192 PMC3525999 23267434

[64] Jeon, H. M. & Lee, J. MET: roles in epithelial-mesenchymal transition and cancer stemness. *Annals Translational Med.* **5**, 5–5 (2017). 10.21037/atm.2016.12.67 PMC5253283 28164090

[65] Kadomatsu, K., Kishida, S. & Tsubota, S. The heparin-binding growth factor midkine: the biological activities and candidate receptors. *J. Biochem.* **153**, 511–521 (2013). 23625998 10.1093/jb/mvt035

[66] Du, R. et al. Matrix metalloproteinase-2 regulates vascular patterning and growth affecting tumor cell survival and invasion in GBM. *Neuro Oncol.* **10**, 254–264 (2008). 18359864 10.1215/15228517-2008-001 PMC2563048

[67] Hagemann, C., Anacker, J., Ernestus, R. I. & Vince, G. H. A complete compilation of matrix metalloproteinase expression in human malignant gliomas. *World J. Clin. Oncol.* **3**, 67–79 (2012). 22582165 10.5306/wjco.v3.i5.67 PMC3349915

[68] Wang, Y. et al. PTCH1 mutation promotes antitumor immunity and the response to immune checkpoint inhibitors in colorectal cancer patients. *Cancer Immunol. Immunother*. **71**, 111–120 (2022). 34028566 10.1007/s00262-021-02966-9 PMC8738454

[69] Horiba, M. et al. Neointima formation in a restenosis model is suppressed in midkine-deficient mice. *J. Clin. Invest.* **105**, 489–495 (2000). 10683378 10.1172/JCI7208 PMC289157

[70] Weckbach, L. T. et al. The cytokine midkine supports neutrophil trafficking during acute inflammation by promoting adhesion via β2 integrins (CD11/CD18). *Blood* **123**, 1887–1896 (2014). 24458438 10.1182/blood-2013-06-510875

[71] Sciumè, G., Santoni, A. & Bernardini, G. Chemokines and glioma: invasion and more. *J. Neuroimmunol.* **224**, 8–12 (2010). 20656128 10.1016/j.jneuroim.2010.05.019

[72] Yeo, E. C. F., Brown, M. P., Gargett, T. & Ebert, L. M. The role of cytokines and chemokines in shaping the immune microenvironment of glioblastoma: implications for immunotherapy. *Cells* **10**, 607 (2021). 33803414 10.3390/cells10030607 PMC8001644

[73] Lachota, M. et al. Mapping the chemotactic landscape in NK cells reveals subset-specific synergistic migratory responses to dual chemokine receptor ligation. *eBioMedicine* 96, (2023). 10.1016/j.ebiom.2023.104811 PMC10520535 37741009

[74] Tibbs, E. J. & Cao, X. Pro-tumoral role of granzyme B to aid in invasion and metastasis. *J. Immunol.* **208**, 178.13 (2022).

[75] Marshall, L. A. et al. Tumors Establish resistance to immunotherapy by regulating Treg recruitment via CCR4. *J. Immunother Cancer*. **8**, e000764 (2020). 33243932 10.1136/jitc-2020-000764 PMC7692993

[76] Korbecki, J., Olbromski, M. & Dzięgiel, P. CCL18 in the progression of cancer. *Int. J. Mol. Sci.* **21**, 7955 (2020). 33114763 10.3390/ijms21217955 PMC7663205

[77] Huang, Y. et al. Microglia/macrophage-derived human CCL18 promotes glioma progression via CCR8-ACP5 axis analyzed in humanized slice model. *Cell. Rep.* **39**, 110670 (2022). 35417708 10.1016/j.celrep.2022.110670

[78] Ravi, V. M. et al. T-cell dysfunction in the glioblastoma microenvironment is mediated by myeloid cells releasing interleukin-10. *Nat. Commun.* **13**, 925 (2022). 35177622 10.1038/s41467-022-28523-1 PMC8854421

[79] Kast, R. E. et al. Glioblastoma-synthesized G-CSF and GM-CSF contribute to growth and immunosuppression: potential therapeutic benefit from dapsone, fenofibrate, and ribavirin. *Tumour Biol.* **39**, 1010428317699797 (2017). 28459367 10.1177/1010428317699797

[80] Kohanbash, G. et al. GM-CSF promotes the immunosuppressive activity of glioma-Infiltrating myeloid cells through interleukin-4 receptor-α. *Cancer Res.* **73** https://doi.org/10.1158/0008-5472.CAN-12–4124 (2013). 10.1158/0008-5472.CAN-12-4124 PMC3829000 24030977

[81] Yang, T., Kong, Z. & Ma, W. PD-1/PD-L1 immune checkpoint inhibitors in glioblastoma: clinical studies, challenges and potential. *Hum Vaccin Immunother*. **17**, 546–553 (2020). 10.1080/21645515.2020.1782692 PMC7899692 32643507

[82] Tibbs, E. & Cao, X. Emerging canonical and Non-Canonical roles of granzyme B in health and disease. *Cancers (Basel)*. **14**, 1436 (2022). 35326588 10.3390/cancers14061436 PMC8946077

[83] Mitamura, T. et al. Induction of anti-VEGF therapy resistance by upregulated expression of Microseminoprotein (MSMP). *Oncogene* **37**, 722–731 (2018). 29059175 10.1038/onc.2017.348 PMC6040890

[84] She, S. et al. PSMP/MSMP promotes hepatic fibrosis through CCR2 and represents a novel therapeutic target. *J. Hepatol.* **72**, 506–518 (2020). 31813573 10.1016/j.jhep.2019.09.033

[85] Maruyama, M. et al. PC3-Secreted microprotein is expressed in glioblastoma Stem-Like cells and human glioma tissues. *Biol. Pharm. Bull.* **44**, 910–919 (2021). 33896885 10.1248/bpb.b20-00868

[86] Gilder, A. S. et al. The urokinase receptor induces a mesenchymal gene expression signature in glioblastoma cells and promotes tumor cell survival in neurospheres. *Sci. Rep.* **8**, 2982 (2018). 29445239 10.1038/s41598-018-21358-1 PMC5813209

[87] Yamada, H., Inazumi, T., Tajima, S., Muramatsu, H. & Muramatsu, T. Stimulation of collagen expression and glycosaminoglycan synthesis by midkine in human skin fibroblasts. *Arch. Dermatol. Res.* **289**, 429–433 (1997). 9248623 10.1007/s004030050216

[88] Gao, Y. F., Zhu, T., Chen, J., Liu, L. & Ouyang, R. Knockdown of collagen α-1(III) inhibits glioma cell proliferation and migration and is regulated by miR128-3p. *Oncol. Lett.* **16**, 1917–1923 (2018). 30008884 10.3892/ol.2018.8830 PMC6036335

[89] Wang, Y. et al. COL1A2 Inhibition suppresses glioblastoma cell proliferation and invasion. *J. Neurosurg.* **138**, 639–648 (2022). 35932265 10.3171/2022.6.JNS22319

[90] Zheng, Y. et al. Expression of β-globin by cancer cells promotes cell survival during blood-borne dissemination. *Nat. Commun.* **8**, 14344 (2017). 28181495 10.1038/ncomms14344 PMC5321792

[91] Onda, M. et al. Decreased expression of haemoglobin beta (HBB) gene in anaplastic thyroid cancer and recovory of its expression inhibits cell growth. *Br. J. Cancer*. **92**, 2216–2224 (2005). 15956966 10.1038/sj.bjc.6602634 PMC2361827

[92] Laurentino, T. S., Soares, R. S., Lerario, A. M., Marie, S. K. N. & Oba-Shinjo, S. M. LOXL3 Silencing affected cell adhesion and invasion in U87MG glioma cells. *Int. J. Mol. Sci.* **22**, 8072 (2021). 34360836 10.3390/ijms22158072 PMC8347215

[93] Liu, C. et al. Exploring the role of LOX family in glioma progression and immune modulation. *Front. Immunol.* **16**, 1512186 (2025). 40270974 10.3389/fimmu.2025.1512186 PMC12014642

[94] Tang, H. et al. Lysyl oxidase drives tumour progression by trapping EGF receptors at the cell surface. *Nat. Commun.* **8**, 14909 (2017). 28416796 10.1038/ncomms14909 PMC5399287

[95] Chang, C. et al. LRP-1 receptor combines EGFR signalling and eHsp90α autocrine to support constitutive breast cancer cell motility in absence of blood supply. *Sci. Rep.* **12**, 12006 (2022). 35835845 10.1038/s41598-022-16161-y PMC9283467

[96] Kadomatsu, K. The midkine family in cancer, inflammation and neural development. *Nagoya J. Med. Sci.* **67**, 71–82 (2005). 17375473

[97] Meng, X. et al. DNA damage repair alterations modulate M2 polarization of microglia to remodel the tumor microenvironment via the p53-mediated MDK expression in glioma. *EBioMedicine* **41**, 185–199 (2019). 30773478 10.1016/j.ebiom.2019.01.067 PMC6442002

[98] Christensen, J. G. et al. Cytoreductive antitumor activity of PF-2341066, a novel inhibitor of anaplastic lymphoma kinase and c-Met, in experimental models of anaplastic large-cell lymphoma. *Mol. Cancer Ther.* **6**, 3314–3322 (2007). 18089725 10.1158/1535-7163.MCT-07-0365

[99] McDermott, U. et al. Genomic alterations of anaplastic lymphoma kinase May sensitize tumors to anaplastic lymphoma kinase inhibitors. *Cancer Res.* **68**, 3389–3395 (2008). 18451166 10.1158/0008-5472.CAN-07-6186

[100] Le Rhun, E. et al. Patterns of response to Crizotinib in recurrent glioblastoma according to ALK and MET molecular profile in two patients. *CNS Oncol.* **4**, 381–386 (2015). 26498130 10.2217/cns.15.30 PMC6083940

[101] Das, A. et al. Synergistic effects of Crizotinib and Temozolomide in experimental FIG-ROS1 Fusion-Positive glioblastoma. *Cancer Growth Metastasis* **8**, 51–60 (2015). CGM.S32801. 10.4137/CGM.S32801 PMC4667559 26648752

[102] Martínez-García, M. et al. Safety and efficacy of Crizotinib in combination with Temozolomide and radiotherapy in patients with newly diagnosed glioblastoma: phase Ib GEINO 1402 trial. *Cancers* **14**, 2393 (2022). 35625997 10.3390/cancers14102393 PMC9139576

[103] Stupp, R. et al. Radiotherapy plus concomitant and adjuvant Temozolomide for glioblastoma. *N. Engl. J. Med.* **352**, 987–996 (2005). 15758009 10.1056/NEJMoa043330

[104] Blakeley, J. O. et al. Phase II study of Iniparib with concurrent chemoradiation in patients with newly diagnosed glioblastoma. *Clin. Cancer Res.* **25**, 73–79 (2019). 30131387 10.1158/1078-0432.CCR-18-0110 PMC6367923

[105] Li, P. et al. A phase II study of anlotinib combined with STUPP regimen in the treatment of patients with newly diagnosed glioblastoma (GBM). *JCO* 39, 2039–2039 (2021).

[106] Roth, P. et al. EORTC 1709/CCTG CE.8: A phase III trial of Marizomib in combination with temozolomide-based radiochemotherapy versus temozolomide-based radiochemotherapy alone in patients with newly diagnosed glioblastoma. *JCO* **39**, 2004–2004 (2021).

[107] Galanis, E. et al. Phase I/II trial of Vorinostat combined with Temozolomide and radiation therapy for newly diagnosed glioblastoma: results of alliance N0874/ABTC 02. *Neuro-Oncology* **20**, 546–556 (2018). 29016887 10.1093/neuonc/nox161 PMC5909661

