# Supplementary material for: Coordinated changes in midkine expression and midkine-associated multiomic profile in glioma microenvironment
Source: Sci Rep. 2025 Aug 20;15:30587. doi: 10.1038/s41598-025-16253-5 (PMC12368106; doi:10.1038/s41598-025-16253-5)
Supplement: Supplementary file 3 — Supplementary Material 3 [file 41598_2025_16253_MOESM3_ESM.docx]

**Coordinated Changes in Midkine Expression and Midkine-associated Multiomic Profile in Glioma Microenvironment**

Mieszko Lachota^1,2^, Katarzyna Zielniok^2^, Agata Góźdź^3^, Patrycja Szpak^3^, Ilona Kalaszczyńska^3^, Radosław Zagożdżon^2^*

^1^ Department of Ophthalmology, Children's Memorial Health Institute, Warsaw, Poland

^2^ Laboratory of Cellular and Genetic Therapies, Medical University of Warsaw, Warsaw, Poland

^3^ Department of Histology and Embryology, Centre for Biostructure Research, Medical University of Warsaw, Warsaw, Poland

**Corresponding author:** Radosław Zagożdżon, Laboratory of Cellular and Genetic Therapies, Medical University of Warsaw, Warsaw, Poland

**E-mail address:** [radoslaw.zagozdzon@wum.edu.pl](mailto:radoslaw.zagozdzon@wum.edu.pl)

**
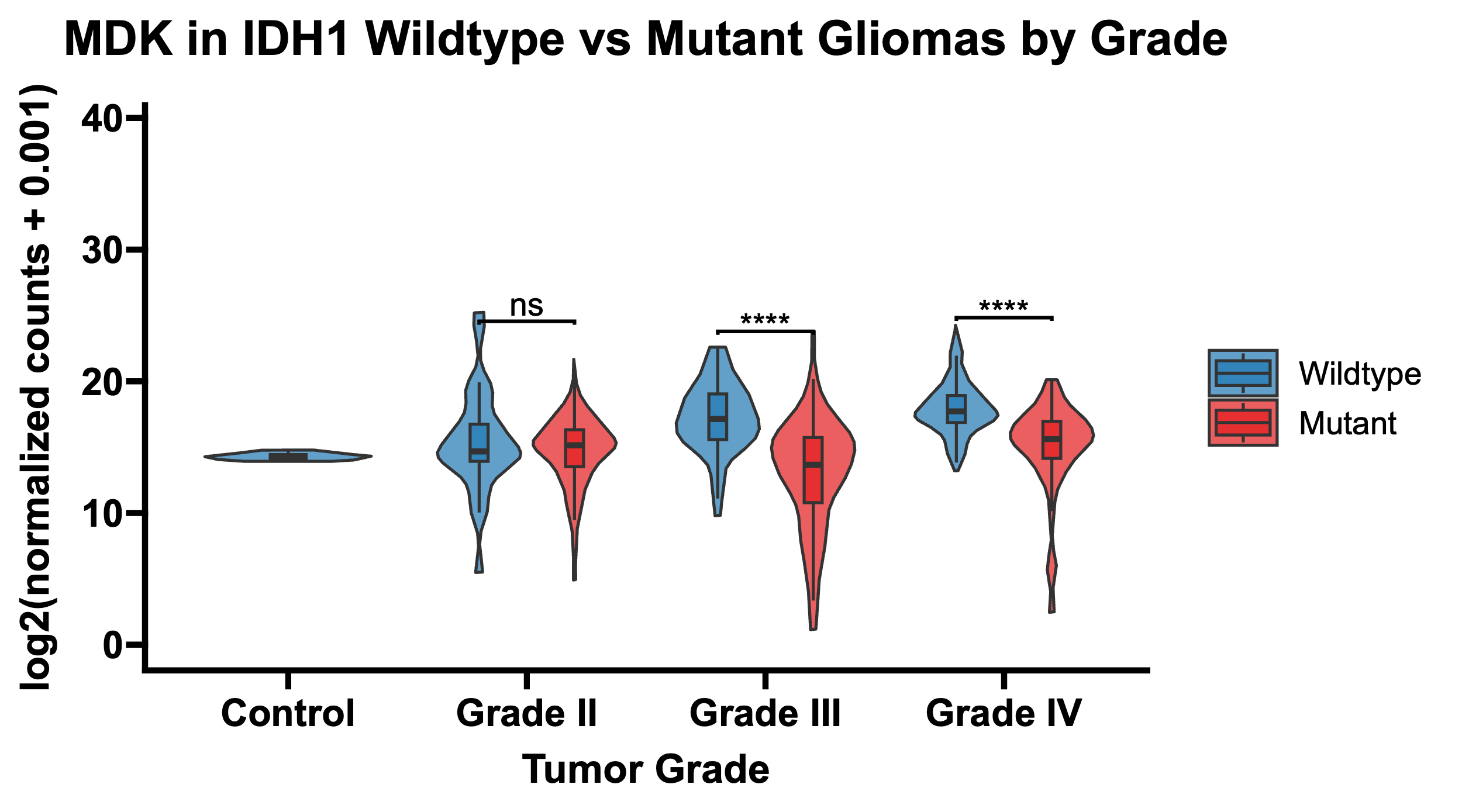
Supplementary Figure 1. *MDK* expression in *IDH1* wild-type and mutant tumors across different glioma grades**

Midkine expression (log2(DESeq2 normalized counts+0.001) in the combined dataset in all gliomas and healthy brain tissues stratified by grade and IDH1 status: wild-type (blue) and mutant (red). Wilcoxon rank-sum test was applied to evaluate the differences between sample types. Significance thresholds: NS p>=0.05; ** p<0.01; *** p<0.001; **** p<0.0001.

| 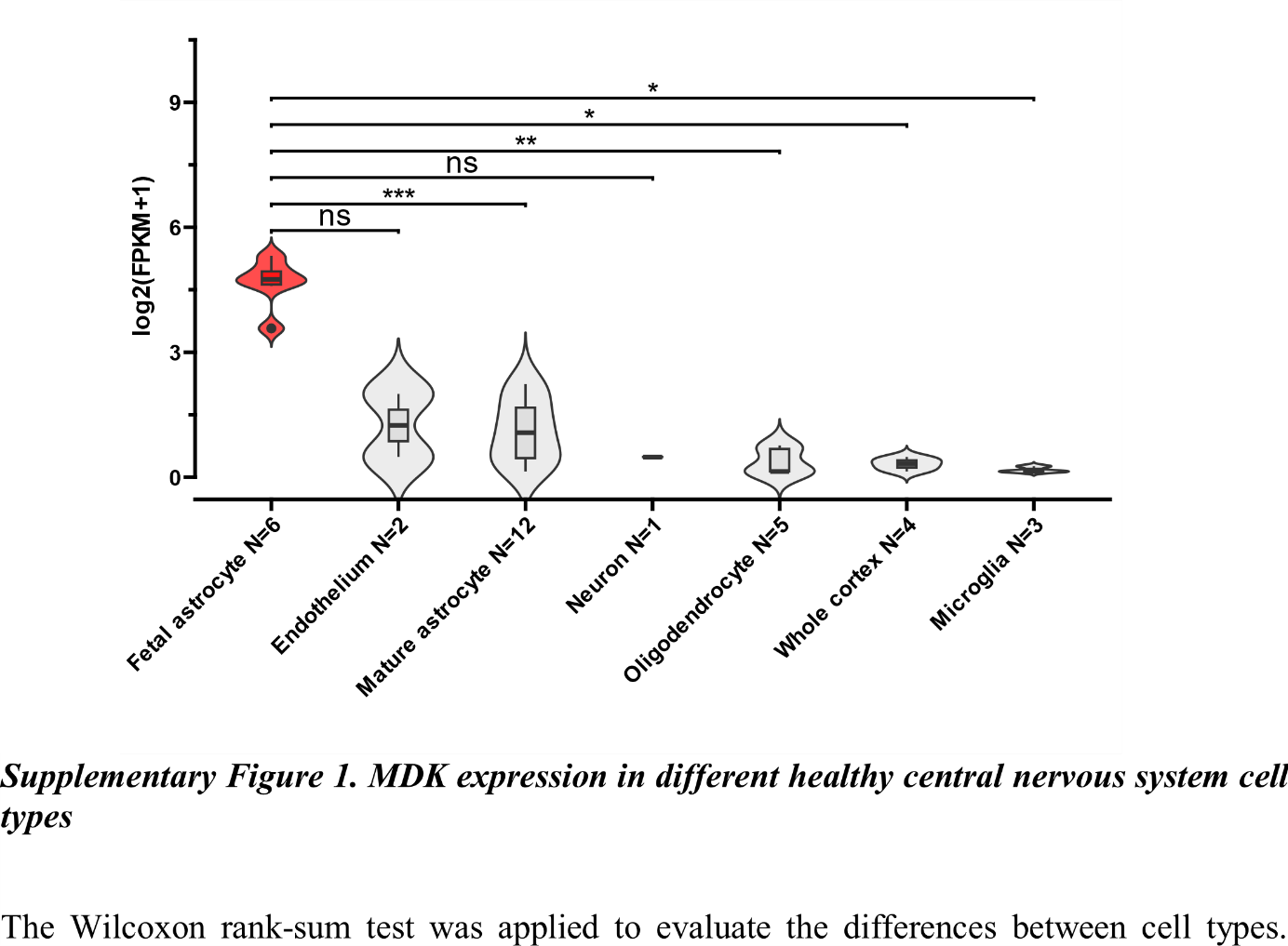 |
| --- |

**Supplementary Figure 2. *MDK* expression in different healthy central nervous system cell types**

Expression of the *MDK* gene log2(FPKM+1) in healthy brain tissues as determined by analyzing expression profiles from sorted brain cell types from 33 samples (<http://www.brainrnaseq.org>) kindly provided by Prof. Steven Sloan. The Wilcoxon rank-sum test was applied to evaluate the differences between cell types. Significance thresholds: (***) p < 0.001; (**) p < 0.01; (*) p < 0.05; (ns) p ≥ 0.05.

**
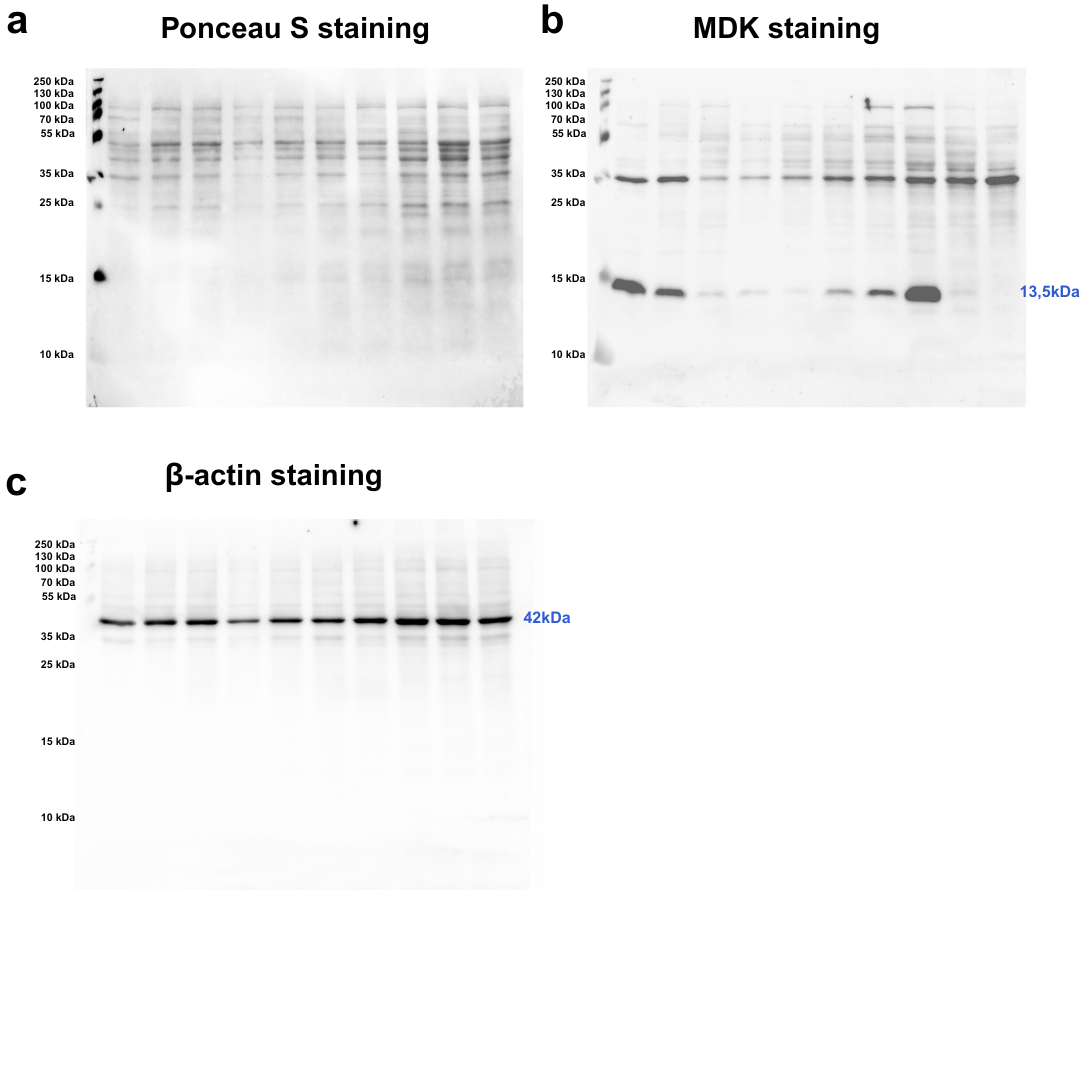
Supplementary Figure 3. Full images of membranes for cropped western blot data in Figure 2b.**

(a) Full image of the blot after Ponceau S staining for the total amount of protein transferred to the membrane. (b) MDK staining by chemiluminescent detection. (c) β-actin staining by chemiluminescent detection.

**
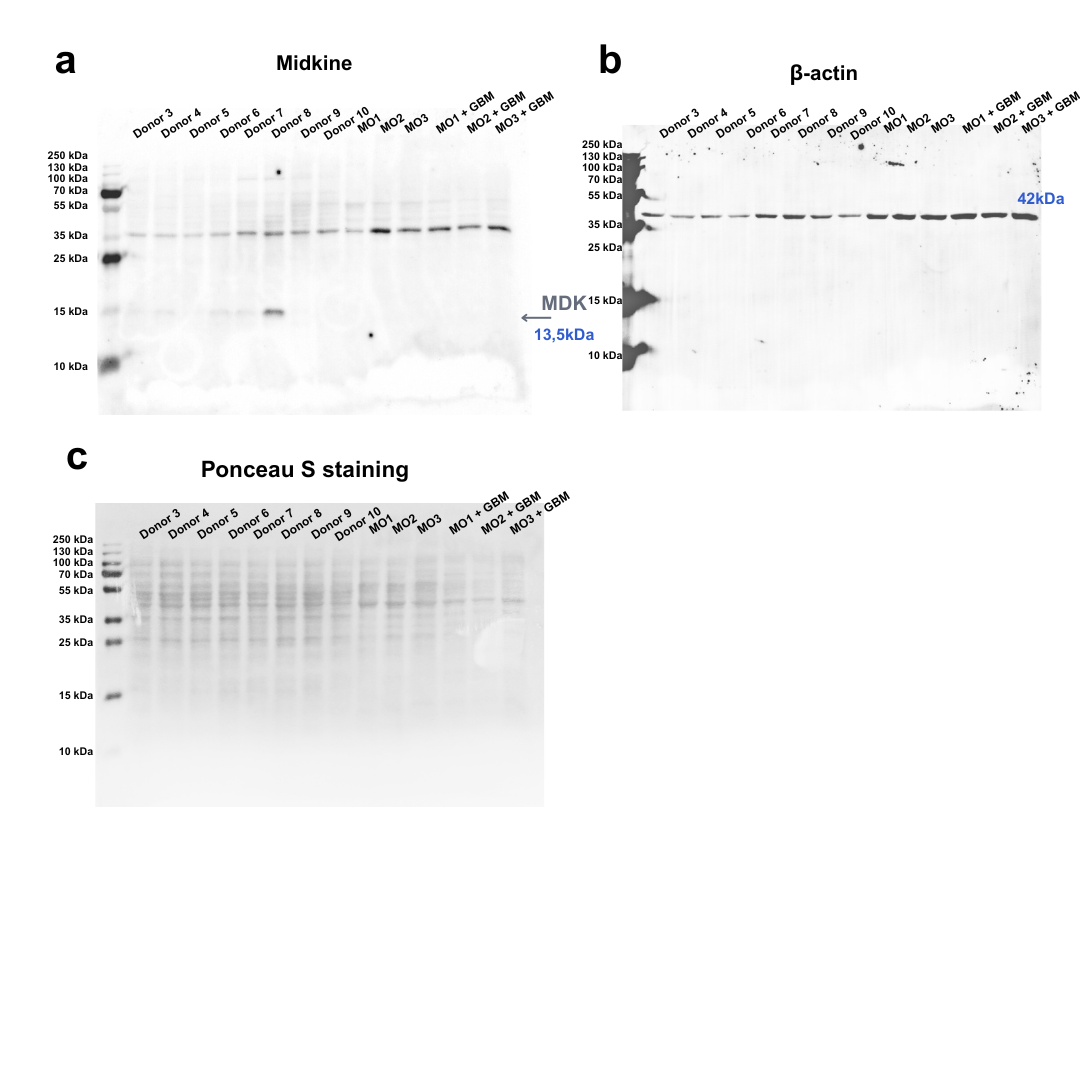
Supplementary Figure 4. Western blot analysis of MDK expression in primary GBM and macrophage cultures.**

Full images of membranes with (a) chemiluminescent staining of MDK, (b) chemiluminescent staining of β-actin, and (c) Ponceau S staining to visualize the total amount of protein transferred to the membrane. The first eight wells are primary GBM populations from 8 donors, the next three are macrophages from three donors cultured in the control medium, and the last three wells are macrophages from the same donors cultured for 72 hours with conditioned medium from GBM cultures.

**
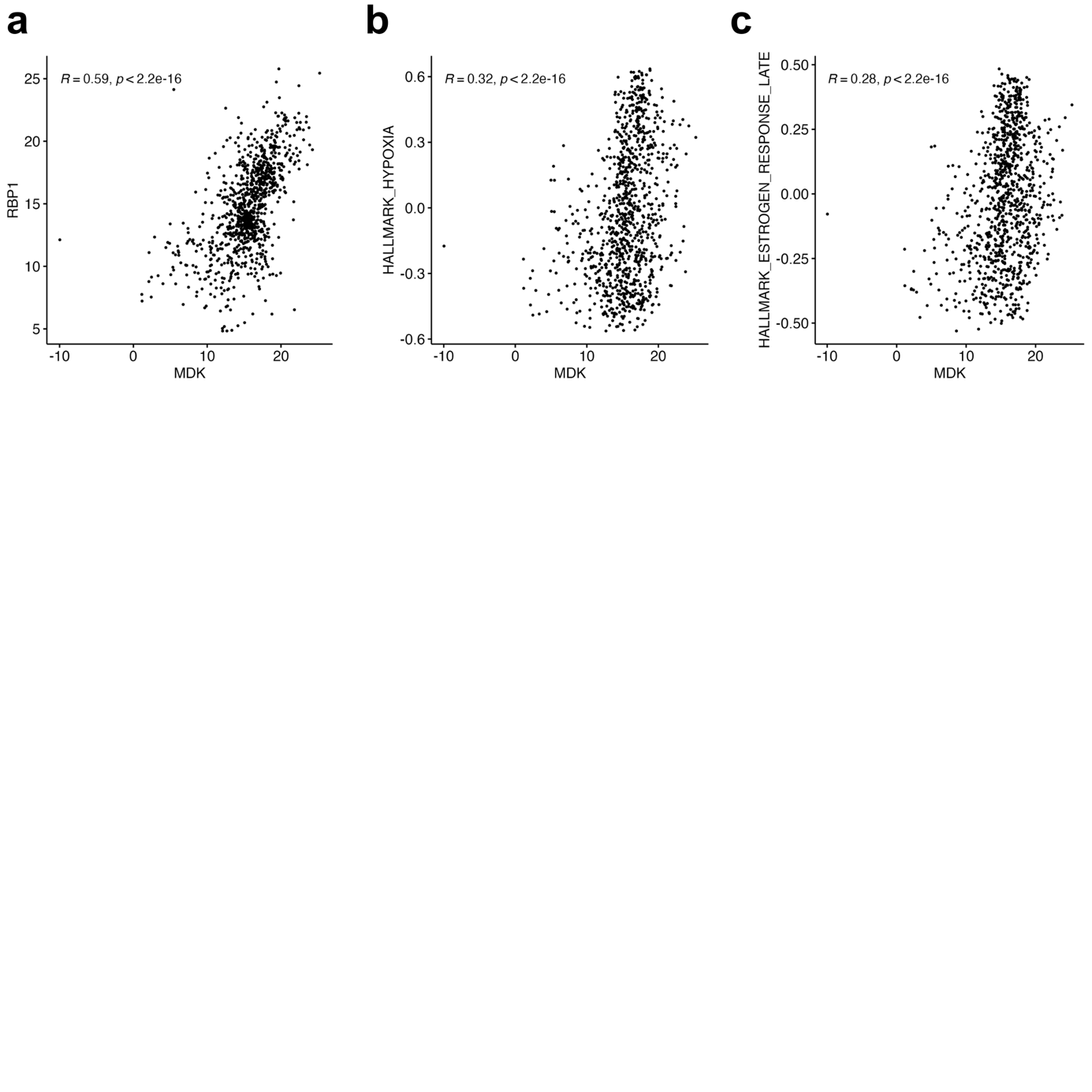
Supplementary Figure 5. Correlation between MDK expression and (a) RBP1 expression (b) HALLMARK_HYPOXIA and (c) HALLMARK_ESTROGEN_RESPONSE_LATE signature scores.**

Displayed expression values are log-transformed (log2(*x* + 0.0001)) normalized counts from the combined dataset of gliomas (N = 993). Healthy tissues were excluded. Spearman's rank correlation was used to assess the relationship between variables.

**
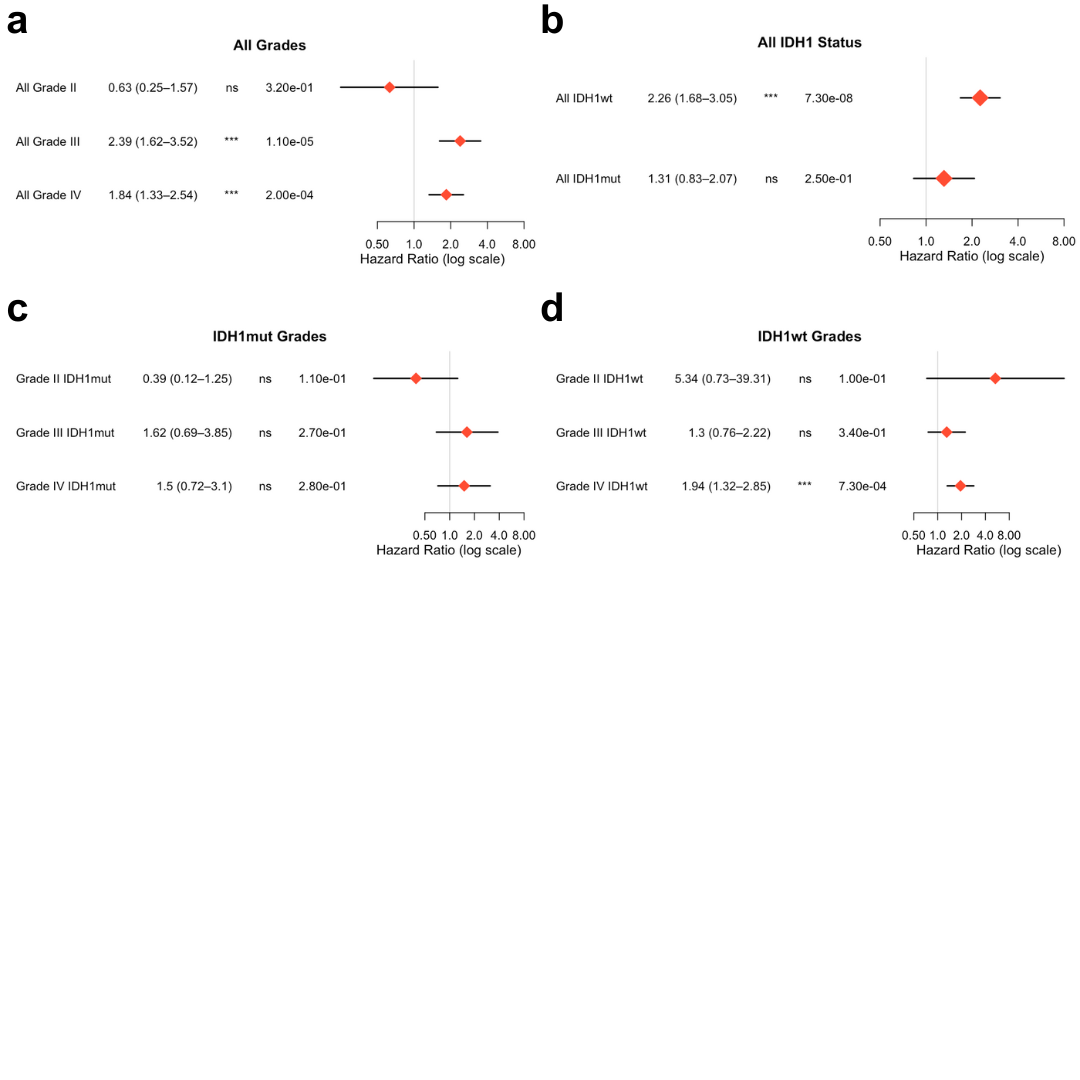
Supplementary Figure 6. Cox analysis of *MDK* prognostic value in *IDH1*^wild-type^ and *IDH1*^mutant^ gliomas**

Forest plot summary of univariate Cox analysis of MDK's prognostic value. (a) the combined dataset is stratified by tumor grade. (b) the combined dataset is stratified by tumor *IDH1* status. (c) the *IDH1*^mutant^ gliomas from the combined dataset are stratified by tumor grade. (d) the *IDH1*^wild-type^ gliomas from the combined dataset are stratified by tumor grade.

**
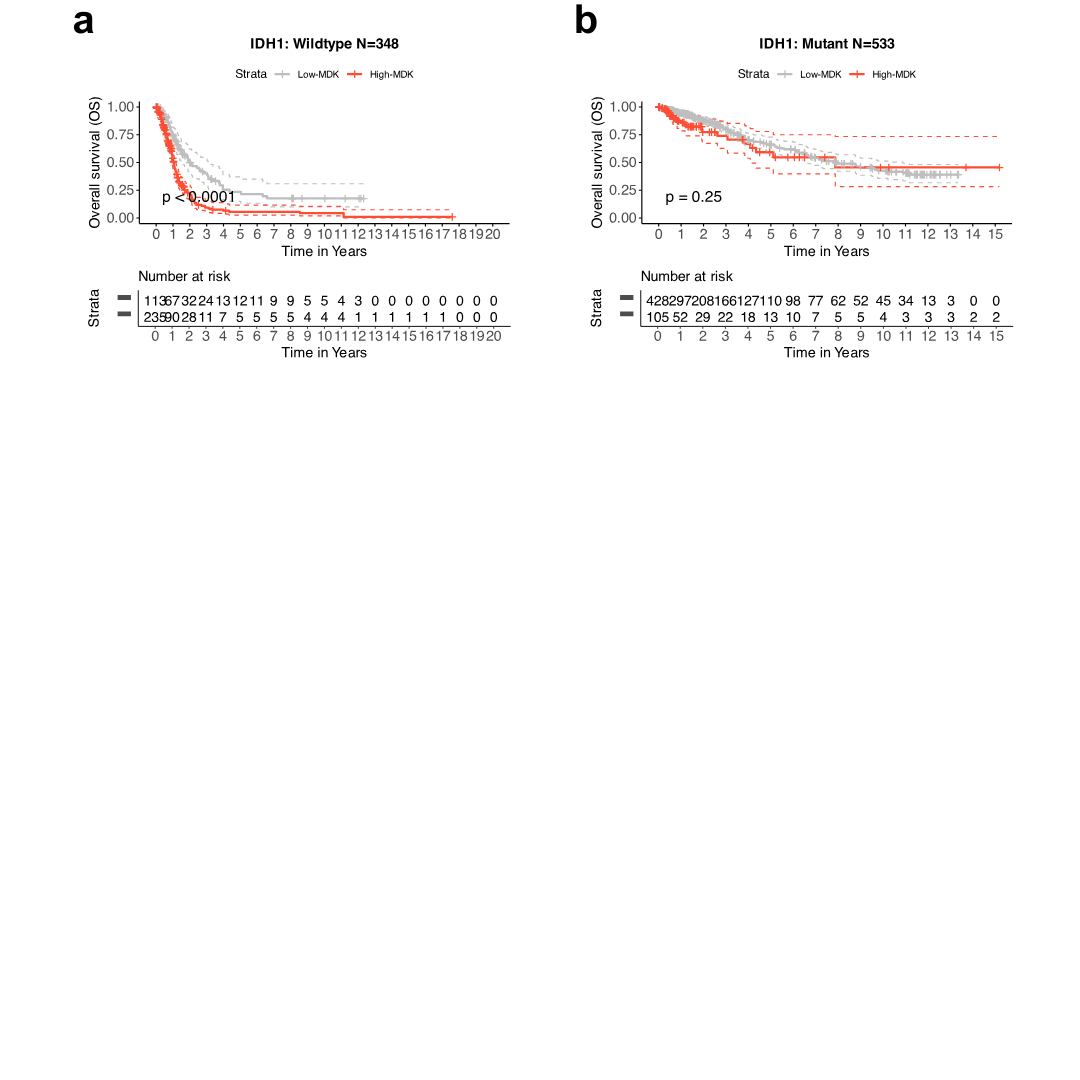
Supplementary Figure 7. Prognostic role of *MDK* in *IDH1*^wild-type^ and *IDH1*^mutant^ gliomas**

Kaplan–Meier survival analysis of overall survival based on MDK expression in combined glioma dataset in (a) *IDH1*^wild-type^ (N =348) and (b) *IDH1*^mutant^ gliomas (N = 533). Below each plot is a 'number at risk' table - the cumulative number of events table and the cumulative number of censored subjects table. A statistical comparison was performed using a log-rank test.

**
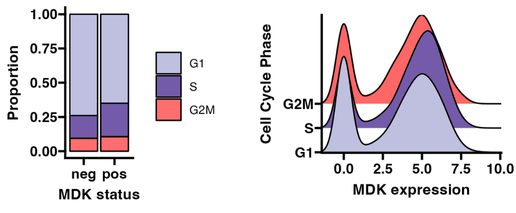
Supplementary Figure 8. Cell cycle analysis of MDK^positive^ and MDK^negative^ cells.**

(a) Proportion of MDK^negative^ and MDK^positive^ glioblastoma cells in G1, S, and G2M cycle phases and (b) expression of MDK in GBM cells in G1, S, and G2M cell cycle phases. We utilized cell cycle markers from Kowalczyk et al. to assign cell cycle state to each malignant cell from the single-cell RNA-sequencing SPC393 dataset. Cell cycle analysis was performed using CellCycleScoring() in Seurat.


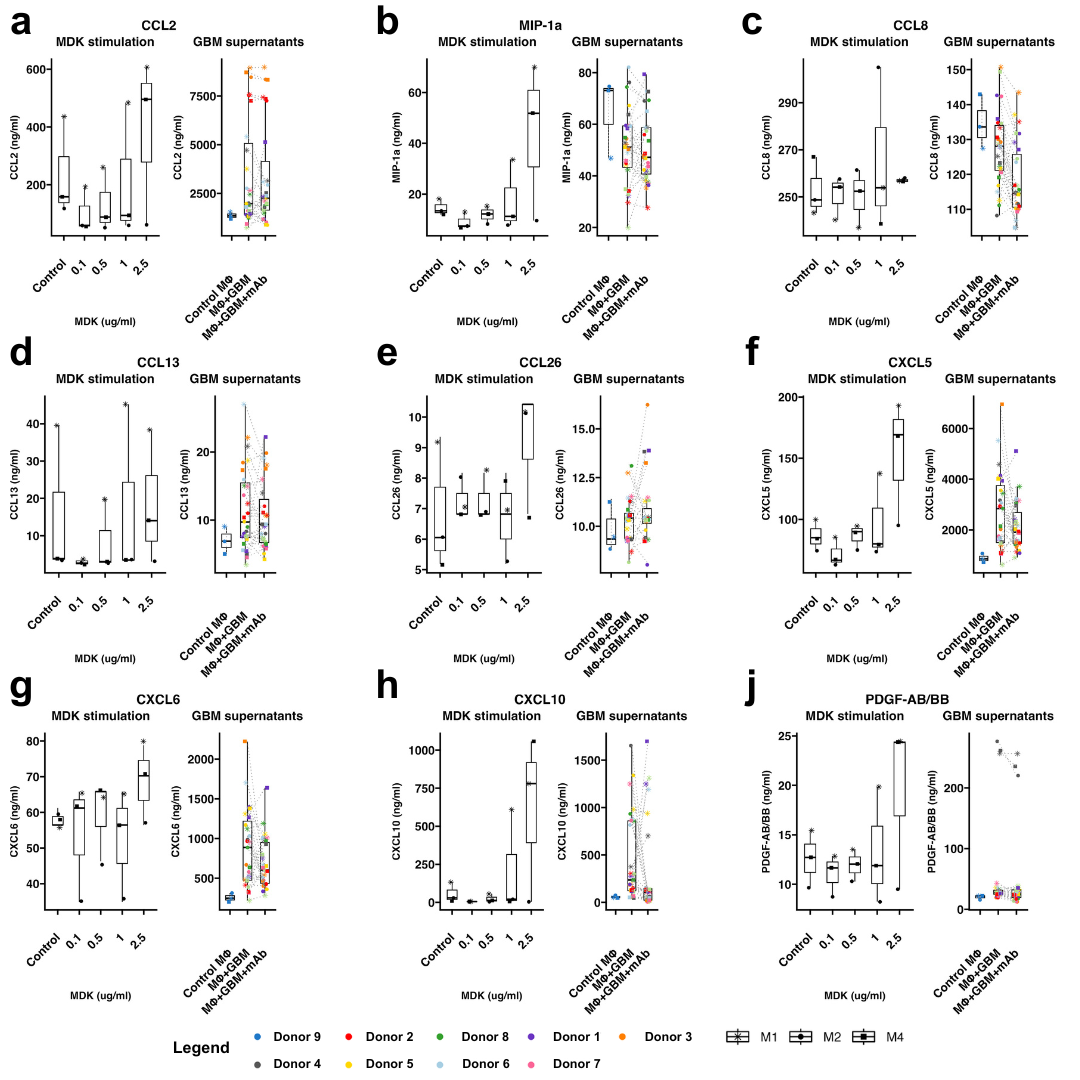
 **Supplementary Figure 9. The effect of midkine on macrophage-secreted chemokines and cytokines**

The macrophages from different donors (N = 3) were stimulated with a control medium and rising physiological concentrations of midkine (0.1, 0.5, 1, and 2.5 ug/ml, the left plot of each panel) or conditioned medium from different primary GBM cell cultures (N = 9) with a neutralizing anti-MDK antibody added (right plot of each panel), with GBM culture medium serving as a control. The secretion of the following analytes was analyzed: (a) CCL2, (b) MIP-1a (CCL3), (c) CCL8, (d) CCL13, (e) CCL26, (f) CXCL5, (g) CXCL6, (h) CXCL10, and (i) PDGF-AA/BB. The results are presented as bar plots with a median and a dot plot with each data point. The concentration of analytes is displayed in ng/ml.


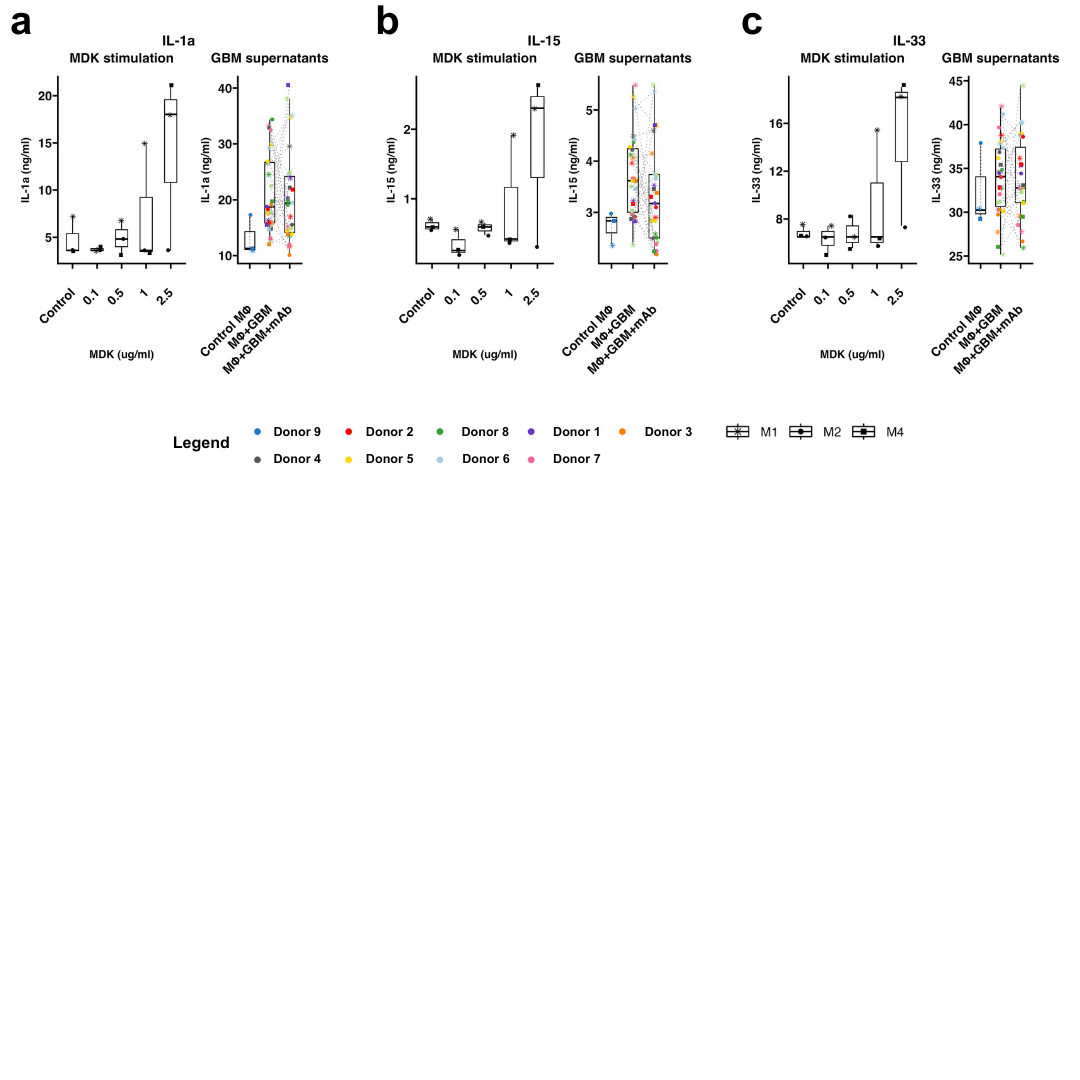
 **Supplementary Figure 10. The effect of midkine on macrophage-secreted interleukins**

The macrophages from different donors (N = 3) were stimulated with a control medium and rising physiological concentrations of midkine (0.1, 0.5, 1, and 2.5 ug/ml, the left plot of each panel) or conditioned medium from different primary GBM cell cultures (N = 9) with a neutralizing anti-MDK antibody added (right plot of each panel), with GBM culture medium serving as a control. The secretion of the following analytes was analyzed: (a) IL-1a, (b) IL-15, and (c) IL-33. The results are presented as bar plots with a median and a dot plot with each data point. The concentration of analytes is displayed in ng/ml.

| **Antibody** | **Supplier** | **Catalog number** | **Dilution factor** |
| --- | --- | --- | --- |
| Anti Midkine recombinant rabbit monoclonal antibody (JF096-5) | Invitrogen, Thermo Scientific | MA5-32538 | 1:1000 |
| Anti-β-actin (C4) | Santa Cruz | Sc-47778 | 1:1000 |
| Anti-rabbit IgG, HRP-linked Antibody | Cell Signaling Technology | 7074P2 | 1:1000 |
| Anti-mouse IgG, HRP-linked Antibody | Cell Signaling Technology | 7076P2 | 1:1000 |

**Supplementary Table 1. List of antibodies utilized for western blotting.**

**Supplementary File 1. A complete list of differentially expressed genes between *MDK*^positive^ (N = 5041 cells) and *MDK*^negative^ (N = 1822 cells) malignant glioblastoma cells in single-cell RNA-seq.**

**Supplementary File 2. A list of differentially expressed genes between *MDK*^high^ (N = 221) and *MDK*^low^ (N = 35) glioblastomas in the combined dataset.**

The results have been filtered by an adjusted p-value threshold of <0.05.
